# Haplotype-resolved genome of *Prunus zhengheensis* provides insight into its evolution and low temperature adaptation in apricot

**DOI:** 10.1093/hr/uhae103

**Published:** 2024-04-08

**Authors:** Wei Tan, Pengyu Zhou, Xiao Huang, Ruyu Liao, Xiaoan Wang, Yaoyao Wu, Zhaojun Ni, Ting Shi, Xiaqing Yu, Huiqin Zhang, Chengdong Ma, Feng Gao, Yufan Ma, Yang Bai, Faisal Hayat, Ouma Kenneth Omondi, Daouda Coulibaly, Zhihong Gao

**Affiliations:** College of Horticulture, Nanjing Agricultural University, Nanjing 210095, China; College of Horticulture, Nanjing Agricultural University, Nanjing 210095, China; College of Horticulture, Nanjing Agricultural University, Nanjing 210095, China; Institute of Fruit, Fujian Academy of Agricultural Sciences, Fuzhou 350013, China; Institute of Fruit, Fujian Academy of Agricultural Sciences, Fuzhou 350013, China; College of Horticulture, Nanjing Agricultural University, Nanjing 210095, China; College of Horticulture, Nanjing Agricultural University, Nanjing 210095, China; College of Horticulture, Nanjing Agricultural University, Nanjing 210095, China; College of Horticulture, Nanjing Agricultural University, Nanjing 210095, China; Institute of Horticulture, Zhejiang Academy of Agricultural Sciences, Hangzhou 310021, China; College of Horticulture, Nanjing Agricultural University, Nanjing 210095, China; College of Horticulture, Nanjing Agricultural University, Nanjing 210095, China; College of Horticulture, Nanjing Agricultural University, Nanjing 210095, China; College of Horticulture, Nanjing Agricultural University, Nanjing 210095, China; Department of Pomology, College of Horticulture, Zhongkai University of Agriculture and Engineering, Guangzhou 510225, China; College of Horticulture, Nanjing Agricultural University, Nanjing 210095, China; Department of Crops, Horticulture and Soils, Faculty of Agriculture, Egerton University, P.O. Box 536, Egerton 20115, Kenya; College of Horticulture, Nanjing Agricultural University, Nanjing 210095, China; Department of Agricultural Sciences and Techniques-Horticulture, Rural Polytechnic Institute for Training and Applied Research (IPR/IFRA) of Katibougou, Koulikoro B.P.224, Mali; College of Horticulture, Nanjing Agricultural University, Nanjing 210095, China

## Abstract

*Prunus zhengheensis*, an extremely rare population of apricots, originated in warm South-East China and is an excellent material for genetic breeding. However, most apricots and two related species (*P. sibirica*, *P*. *mandshurica*) are found in the cold northern regions in China and the mechanism of their distribution is still unclear. In addition, the classification status of *P. zhengheensis* is controversial. Thus, we generated a high-quality haplotype-resolved genome for *P*. *zhengheensis*, exploring key genetic variations in its adaptation and the causes of phylogenetic incongruence. We found extensive phylogenetic discordances between the nuclear and organelle phylogenies of *P*. *zhengheensis*, which could be explained by incomplete lineage sorting. A 242.22-Mb pan-genome of the *Armeniaca* section was developed with 13 chromosomal genomes. Importantly, we identified a 566-bp insertion in the promoter of the *HSFA1d* gene in apricot and showed that the activity of the *HSFA1d* promoter increased under low temperatures. In addition, *HSFA1d* overexpression in *Arabidopsis thaliana* indicated that *HSFA1d* positively regulated plant growth under chilling. Therefore, we hypothesized that the insertion in the promoter of *HSFA1d* in apricot improved its low-temperature adaptation, allowing it to thrive in relatively cold locations. The findings help explain the weather adaptability of *Armeniaca* plants.

## Introduction

Apricot, scientifically known as *Prunus armeniaca* L., belongs to the *Armeniaca* section of the genus *Prunus*, family Rosaceae. This deciduous tree is cultivated for its edible fruits and yields, with over 4.1 million tonnes of fruit produced globally each year [1]. In China, apricots are popular not only for their excellent taste but also for their lovely blooms, which have earned the acclaim of many poets. Apricot agriculture has a long history in China, with indications of apricot cultivation dating back 3000–4000 years [2]. Cultivated apricots are widely distributed in China but are mainly concentrated in the north and scattered in the south. However, both ancient and modern documents clearly indicate that there is no distribution of apricots in Fujian or Taiwan [3]. Two other closely related species of apricots, *P*. *sibirica* L. and *P*. *mandshurica* (Maxim.) Koehne, are also distributed in northern China, such as Heilongjiang and Liaoning provinces [4]. In 1996, Zhang *et al*. first discovered a species in Chouling Mountain, Waitun Township, Zhenghe County, Nanping City, Fujian Province [5]. This germplasm,named *Prunus zhengheensis*, has remarkably similar characteristics to apricot, especially its blooms and fruits, and has been defined as a new species in the *Armeniaca* section. *Prunus zhengheensis* has a very small and precious population endemic to China, and it has been included in the list of China’s key protected wild plants. At present, the population of this species is less than 30 individuals, including 6 plants in Zhenghe County, Fujian, and 17 plants in Qingyuan County, Zhejiang. Other species in the *Armeniaca* section, *Prunus mume* and *P. hongpingensis*, are related to apricot, and its wild varieties are widely distributed in southern China, overlapping with the distribution of *P*. *zhengheensis*. Even though *P*. *zhengheensis* and apricots are quite similar in morphology, researchers have suggested that *P*. *zhengheensis* is more closely related to *P. mume*. The ITS sequence and chloroplast genome phylogeny show that *P*. *zhengheensis* is more closely related to *P. mume* than to apricot [6–8]. However, the cause of the classification inconsistency is unclear.

With the advancement of sequencing technology, an increasing number of plant genomes have been successfully sequenced. On the one hand, phylogeny based on genomics can more accurately reconstruct the species tree, resolving a long-standing dilemma for taxonomists. On the other hand, a sufficient number of genomes can be utilized to construct a pan-genome, which can capture sequences influenced by structural variations (SVs) as well as sequences that may not be present in a single individual's reference sequence [9]. SVs contribute significantly to genetic diversity and influence phenotypic variation [10]. In *Prunus persica*, a 487-bp deletion in the promoter of *PpMYB10*.*1*, for example, was linked to the colour of the flesh around the stone [11]. In several crops, such as *Arachis hypogaea* [12], *Pennisetum glaucum* [13], *Brassica napus* [14] and *Solanum lycopersicum* [15], studies have shown that SVs have a significant impact on numerous genes related to environmental stress responses. Previously, limitations in the techniques and methods used to analyse SVs have hindered our understanding of the extent and significance of these changes. With improvements in the quality of plant genomes, the study of plant SVs has gained popularity in recent years. In particular, the identification of SVs based on a graphical pan-genome has become an advanced method for their study. To date, a total of 10 genomes from four species in the *Armeniaca* section have published genomes at the chromosomal level: *P. mume* (one wild and one cultivar), apricot (six cultivars), *P*. *sibirica* and *P*. *mandshurica*. Recently, the genomes of *P. zhengheensis* and *P. hongpingensis* were also published [16]. This gives us a valuable database to build the *Armeniaca* pan-genome. The reason why apricot is distributed in the frigid north when *P*. *zhengheensis* originated in South China can be investigated by SV identification based on the *Armeniaca* pan-genome.

Temperature has a significant influence on plant growth at various developmental stages and restricts plant geographical dispersion [17, 18]. Heat shock transcription factors (HSFs) are prevalent in eukaryotes. Plant HSFs can be categorized into three types based on their structure, namely *HSFA*, *HSFB* and *HSFC*, which are important in plant responses to extreme temperature stress (high or low temperatures) [19]. The *HSFA1* group contains four genes: *HSFA1a*, *HSFA1b*, *HSFA1d* and *HSFA1e*, which together regulate >65% of all genes induced by high-temperature stress, and *HSFA1a*, *HSFA1b* and *HSFA1d* are the master regulators of heat stress response in *Arabidopsis thaliana* [20, 21]. Tomato plants overexpressing *HSFA1a* and *Glycine max* plants overexpressing *HSFA1* exhibit increased thermotolerance [22]. In pepper (*Capsicum annuum* L.), *CaHSFA1d* improves plant thermotolerance by maintaining H_2_O_2_ homeostasis [23]. Pea (*Pisum sativum* L.) plants overexpressing *HSFA1d* from *A. thaliana* have an improved reactive oxygen scavenging system to cope with heat stress [24]. In addition to high temperatures, *HSFA* genes are also involved in the response to low temperatures. For example, *HSFA1d* promotes hypocotyl elongation under chilling stress by enhancing the expression of ribosomal protein genes in *A. thaliana* [17]. In cucumber, the *HSF* gene *CsHSFA1d* can be induced by both heat shock and low temperatures [25].

In this study we report the first haplotype-resolved chromosome-level genome assemblies of *P*. *zhengheensis*. Based on this high-quality genome, we verified that incomplete lineage sorting (ILS) contributed to the conflicting phylogenetic relationships between nuclear and organelle phylogenies. Together with the *P*. *zhengheensis* genome, we built a graph-based pan-genome and performed SV calling, empowering the identification of variants potentially associated with apricot low-temperature adaptation and accelerating the breeding of apricots.

## Results

### Genome assembly and annotation

**Figure 1:**
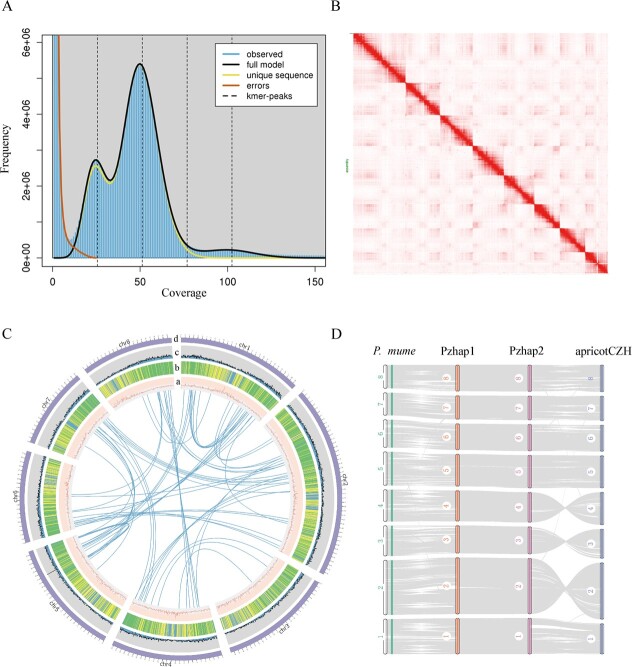
Genomic characterization of *P. zhengheensis* and collinearity analysis of related species. **A** Genome survey of *P*. *zhengheensis*. **B** Genome-wide Hi-C map showing the interaction frequency distribution of Hi-C links among the chromosomes of *P*. *zhengheensis*. **C** From a to d: GC content, repeats, gene count, and chromosomes; in the circle are gene links. (D) Collinearity analysis of two haplotype genomes of *P*. *zhengheensis* (Pzhap1 and Pzhap2), apricotCZH and *P. mume*.

The genome of *P. zhengheensis* was sequenced and assembled. Based on 16.04 Gb Illumina short reads, a *k*-mer analysis showed that the genome size of *P*. *zhengheensis* was ~241.94 Mb, with a 1.00% level of heterozygosity and 41.86% repeat sequences (Fig. 1A). Flow cytometry was used to confirm that *P*. *zhengheensis* was diploid (Supplementary Data Fig. S1). A total of 12.22 Gb consensus (CCS) reads and 31.11 Gb Hi-C data (Supplementary Data Table S1) were used to assemble two haplotype-resolved genomes: haplotype 1 (hap1) and haplotype 2 (hap2). Plion v1.24 was then employed to correct the assembly results, and Purge_dups v1.2.5 software was used to remove redundancies. Subsequently, chromosome mounts were performed using Hi-C data to obtain the final assembly, including eight pseudochromosomes in the haplotype genome (Fig. 1B and C). The genome size of hap1 was 241.37 Mb and that of contig N50 was 25.93 Mb. The scaffold N50 was 21.24 Mb, with a mount rate of 92%. The total length of hap2 was 235.57 Mb, with an N50 size of 16.52 Mb. The results of mapping the Illumina data to the final genome indicated that the coverage for hap1 and hap2 was 97.8 and 97.7%, respectively, highlighting the accuracy and high integrity of the genome assembly.

**Table 1:** Genome assembly statistics for *Prunus zhengheensis*.

	**hap1**	**hap2**
Total length (bp)	241 369 245	235 573 950
Total number	46	39
Scaffold N50 (bp)	21 242 234	16 520 266
Contig N90 (bp)	9 432 709	5 619 964
Average (bp)	5 247 157.50	6 040 357.69
Maximum (bp)	30 595 378	28 518 252
GC%	37.83	37.77
Repeat content (%)	45.40	44.31
Protein-coding genes	25 986	26 295
BUSCO completeness (%)	93.6	92.6
Complete and single-copy BUSCOs (%)	91.6	91.3

The repetition content predicted for hap1 and hap2 was 45.40% (109.59 Mb) and 44.31% (104.39 Mb), respectively (Supplementary Data Table S2). By combining homology-based and *de novo* gene prediction, as well as RNA-seq data, we identified 25 986 genes in hap1 and 26 295 genes in hap2. The assessment of predicted genes using BUSCO software revealed 93.6 and 92.6% gene completeness in hap1 and hap2, respectively. For functional annotation, 23 393 and 23 676 genes were found in hap1 and hap2, respectively, matching entries in the Gene Ontology (GO), Kyoto Encyclopedia of Genes and Genomes (KEGG), and PFAM databases. The two haplotype genomes of *P*. *zhengheensis* exhibited strong collinearity with apricotCZH (*P. armeniaca* ‘Chuanzhihong’) and *P. mume* (Fig. 1D), further verifying the genome’s accuracy. In our study, hap1 was used for the follow-up analysis, unless otherwise noted.

### Discordances in *P*. *zhengheensis* topology between nuclear and organelle genomes were caused by incomplete lineage sorting

**Figure 2:**
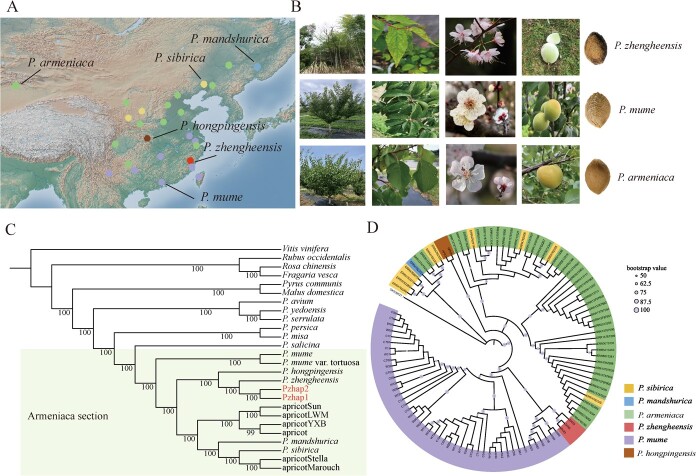
Geographical distribution, morphological comparison and phylogenetic analysis of *P. zhengheensis* with *P. mume* and apricot. **A** Geographical distribution map of *P*. *zhengheensis*, wild apricot, cultivated apricot, *P*. *mandshurica*, *P*. *sibirica*, wild *P. mume* and cultivated *P. mume*. **B** Morphological characteristics of *P*. *zhengheensis*, *P. mume* and apricot. **C** Phylogenetic analysis of *P*. *zhengheensis* based on 151 single-copy genes. *P. armeniaca* ‘Jintaiyang’, *P. armeniaca* ‘Longwangmao’, *P. armeniaca* ‘Yinxiangbai’, *P. armeniaca* ‘Chuanzhihong’, *P. armeniaca* ‘Stella’ and *P. armeniaca* accession ‘Marouch #14’ are represented by apricotSun, apricotLWM, apricotYXB, apricotCZH, apricotStella and apricotMarouch, respectively. **D** Phylogenetic analysis of *P*. *zhengheensis* based on chloroplast genomes.

To investigate the taxonomic status of apricots, single-copy genes from 23 genomes were selected to reconstruct phylogenetic trees. The *Armeniaca* section was placed in one branch, and *P*. *zhengheensis* was more closely related to *P. hongpingensis*, the common apricot (cultivated species of *P. armeniaca*) and wild *Armeniaca* (*P*. *sibirica* and *P*. *mandshurica*) than to *P. mume*. Moreover, we discovered that *P. zhengheensis* and *P. hongpingensis* exhibited the closest genetic relationship. Following the use of the nuclear genome to establish the evolutionary relationships of *P*. *zhengheensis*, organelle genomes were utilized to examine the phylogeny of *P*. *zhengheensis*, which was previously widely employed for eukaryote phylogeny. Phylogenetic trees based on chloroplast and mitochondrial genomes showed that *P*. *zhengheensis* and *P. mume* were clustered in one branch (Fig. 2D and Supplementary Data Fig. S2). Conflicting topologies of organelle genomes and nuclear genomes may result from stochastic effects caused by a small sample size, ILS and introgression [26]. To eliminate stochastic effects due to the small sample size, we successfully assembled 3 *P*. *zhengheensis*, 2 *P. hongpingensis*, 45 *P. armeniaca*, 1 *P*. *mandshurica*, 10 *P*. *sibirica* and 46 *P. mume* chloroplast genomes from previous studies for phylogenetic analysis and chloroplast haplotype construction (Fig. 2A). Additionally, *P*. *zhengheensis* and *P. mume* (from Jiangsu cultivars and Yunan wild samples) clustered together in one branch (Fig. 2B). Chloroplast haplotype analysis revealed that *P*. *zhengheensis* was related to hap3, hap7, hap17, hap18, hap19 and hap20, and these haplotypes were from Jiangsu (hap3, hap7) and Yunnan (hap17, hap18, hap19, hap20) in the chloroplast phylogenetic tree (Supplementary Data Fig. S3 and Fig. 2D).

To distinguish introgression from ILS, we performed quantifying introgression via branch length (QuIBL) analysis on 60 topologies, with apple as an outgroup. Since the bayesian information criterion (BIC) value was <0, when ΔBIC (ΔBIC = BIC2Dist − BIC1Dist) was >10 the scenario of ILS only with a lower BIC value was preferable. However, when ΔBIC was less than −10 the scenario of a mixture of ILS and introgression with a lower BIC value was preferable. In other cases, the two scenarios were indistinguishable [27]. Of all tested triplets, 28.33% showed significant evidence of introgression (17 of 60 triplets). The topology of (apricot, Pzhap1) *P. mume* had 761 loci, accounting for 52% of all loci (1474) and supporting the species topology that Pzhap1 was more closely related to apricot. We did not consider the topologies of individual trees in (apricot, Pzhap1) *P. mume* when assessing discordances because they corresponded to the species tree. The discordant topology with the species tree (*P. mume*, Pzhap1) apricot was likely ILS, as the ILS proportion was high (90.45%) and ΔBIC >10. Similarly, another discordant topology (*P. mume*, apricot) for Pzhap1 was also likely ILS, with ΔBIC >10. Overall, we estimated that the discordant topologies were attributed to 90.52% ILS loci (645 of 713 loci discordant with the species tree), suggesting that ILS was the main reason for the conflicting phylogeny of *P*. *zhengheensis* (Supplementary Data Table S3).

### Transposable element insertion history of *Armeniaca*

**Figure 3:**
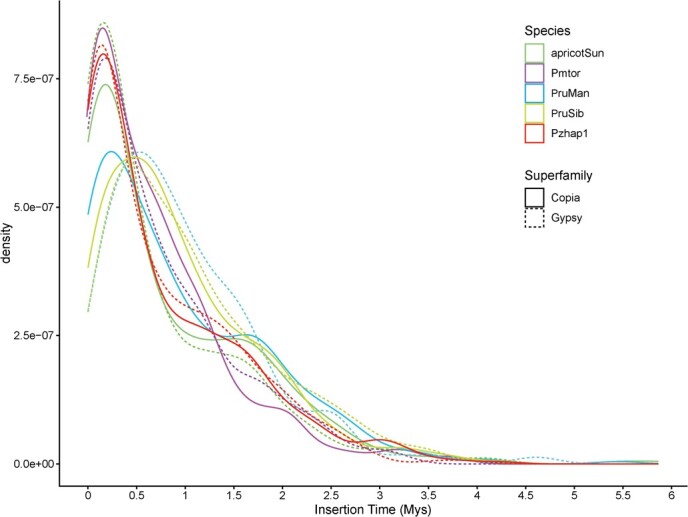
Estimated insertion time of full-length LTR retrotransposons in five *Armeniaca* species. The *X* axis represents insertion time and the *Y* axis represents density. *Prunus sibirica* is represented by PruSib, *P*. *mandshurica* by PrunMan and *P. mume* var. *tortuosa* by Pmtor.

Transposable elements (TEs) are genetic sequences that mediate their own replication and integration into host genomes from all domains of life. Long terminal repeat retrotransposons (LTRs) are Class I TEs that play a significant role in genome structure development, gene function expression and genome structure evolution in higher plant genomes [28]. Here we investigated the insertion time of full-length LTRs in the genomes of five representative *Armeniaca* species. An estimation of the TE insertion time indicated that LTR expansion was relatively recent in apricot, *P. mume* and *P*. *zhengheensis*, around 0.25 MYA, whereas a relatively more ancient LTR expansion occurred in *P*. *sibirica* in the Copia and Gypsy superfamily, around 0.5 MYA (Fig. 3). Interestingly, the Copia superfamily in *P*. *mandshurica* was expanded relatively recently (0.25 MYA), but Gypsy expansion was relatively ancient (0.5 MYA) (Fig. 3).

### Pan-genome of *Armeniaca* species

**Figure 4:**
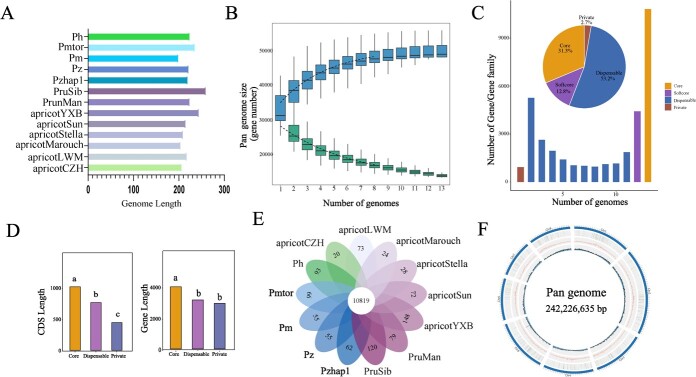
Pan-genome analysis of the *Armeniaca* section. **A** Total chromosome length of 13 *Armeniaca* genomes. *Prunus sibirica* is represented by PruSib, *P*. *mandshurica* by PrunMan, *P. mume* var. *tortuosa* by Pmtor, *P. mume* by Pm, *P. hongpingensis* by Ph and *P*. *zhengheensis*, published recently, by Pz. **B** Influence of the number of *Armeniaca* genomes on the number of pan and core gene families. **C** Characteristics of each individual genome and the pan-genome. The histogram depicts the number of gene families in various genomes. The percentage of gene families in each category is displayed in the pie chart. Core gene families are found in all genomes. Softcore families are gene families that make up >90% of the genome. Private gene families are present in a single genome. The remaining gene families are assigned to dispensable gene families. **D** CDS lengths and gene lengths in core, dispensable and private gene families. **E** Petal map of specific gene families in 13 *Armeniaca* genomes. **F** Graph-based pan-genome of 13 *Armeniaca* genomes. From the inside out: GC content, repeats, gene count, chromosomes.

We utilized six *de novo*-assembled genome sequences of cultivated apricot, two wild species of *Armeniaca* (*P*. *mandshurica* and *P*. *sibirica*), two *P. mume* individuals (one cultivated and one wild) and *P*. *zhengheensis* assembled in this study, along with *P. zhengheensis* and *P. hongpingensis*, which have been published recently for pan-genome construction. The length of the 13 *Armeniaca* genomes ranged from 198.86 to 259.44 Mb. Among them, the *P*. *sibirica* genome was the largest, while the genome of *P. mume* (wild) was the smallest (Fig. 4A). For the family-based pan-genome, as more genomes were progressively included in the analysis the total number of gene families consistently increased until it eventually levelled off. These findings suggest that the pan-genome largely encompassed the majority of the genes found within the *Armeniaca* section, as illustrated in Fig. 4B. Of the gene families (core and softcore gene families), 44.1% were present in >11 genomes, 53.2% in 2–11 genomes (dispensable gene families), and 2.7% in a single genome (private gene families) (Fig. 4C). The coding sequence (CDS) lengths and gene lengths of the core gene families were significantly longer than those of the other types of gene families (dispensable gene families and private), which was consistent with previous studies (Fig. 4D) [9]. In addition, we identified 34 specific gene families (including 1731 genes) of *P*. *zhengheensis* (combined with Pzhap1 and Pz) relative to other related *Armeniaca* species (Fig. 4E). Functional annotation indicated that these genes were mainly involved in environmental adaptability, carbohydrate metabolism and signal transduction (Supplementary Data Table S4).

The 13 *Armeniaca* genomes were used to construct a graph-based pan-genome, with *P*. *zhengheensis* serving as the principal reference. The total length of the pan-genome was 242 226 635 bp, containing eight chromosomes and 30 702 genes (Fig. 4F). Through whole-genome comparisons, we identified 4757 novel sequences inserted into the pan-genome, of which 2473 sequences were further categorized as potential translocation. The mean, minimum, maximum and sum of novel sequence lengths were 5421, 1002, 41 329 and 25 786 959 bp, respectively. A total of 20 044 presence/absence variations were obtained, with 4776 from apricotCZH, 1568 from apricotYXB, 2374 from apricotMarouch, 1507 from apricotStella, 1865 from *P*. *mandshurica*, 3715 from *P. mume*, 2054 from *P. mume* var. *tortuosa*, and 2185 from *P. hongpingensis*. These results suggest that the graph-based pan-genome could offer a useful platform for the analysis of critical agronomic traits in the *Armeniaca* section.

### Structural variation in *Armeniaca*

**Figure 5:**
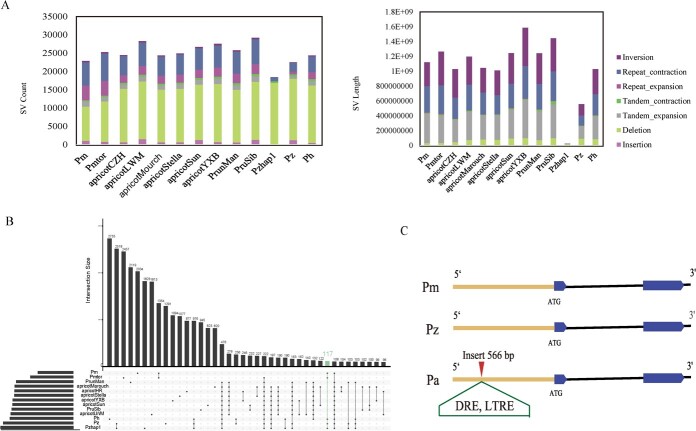
Structural variation in 13 *Armeniaca* genomes. **A** Counts and sequence lengths of seven SV types in 13 *Armeniaca* genomes. **B** Deletion SVs of 13 genomes in a Venn diagram. **C** The promoter of the *HSFA1*d gene had a 566­ bp insertion that was absent in *P*. *zhengheensis* and *P. mume*. DRE and LTRE are *cis*-acting elements associated with low temperature and present in SV fragments.

To identify SVs, all 13 genomes were realigned to the pan-genome using MUMmer v4.00, and SVs were derived from alignments using Assemblytics v1.2.1. Assemblytics software divided SVs into seven types, including insertion, deletion, tandem expansion, tandem contraction, repeat expansion, repeat contraction and inversion. Among these, the number of deletion SVs was the largest, ranging from 9367 to 16 865 per genome. We observed an average 18-fold excess number of deletions versus the number of insertions, amounting to an average 71-fold excess of bases in deletions versus bases in insertions (Fig. 5A and Supplementary Data Table S5).

**Figure 6:**
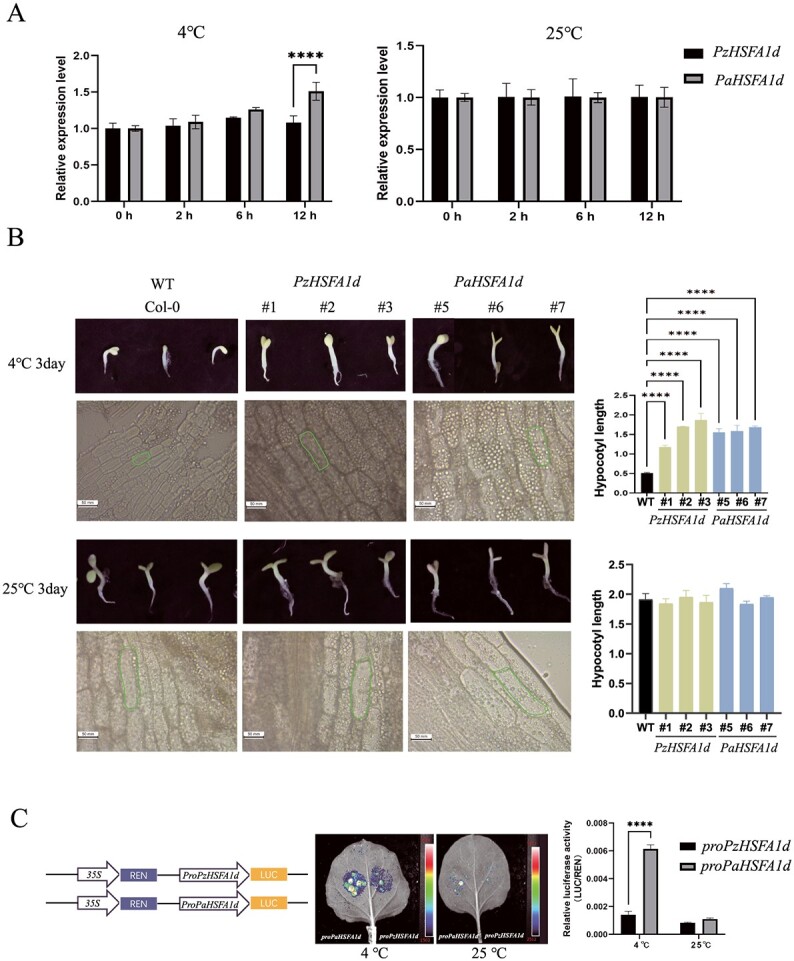
*HSFA1d* gene function research. **A** Relative RNA expression level of the *HSFA1d* gene in apricot and *P. zhengheensis* after 4 and 25°C treatment for 12 h. **B***HSFA1d* promoted hypocotyl growth under chilling in overexpressing *A. thaliana*. **C** Transient expression assay of luminescence intensity showing the transcription activity of promoters of *PzHSFA1d* and *PaHSFA1d* (^****^ Pvalue < 0.0001).

The deletion types of SVs were used for further investigation. The deletion SVs of 13 genomes were extracted, and the deletion intersections of each species were plotted in a Venn diagram (Fig. 5B). Considering that *P*. *zhengheensis*, *P. hongpingensis* and *P. mume* had overlapping ecological zones, we used the deletion regions occurring in *P*. *zhengheensis*, *P. hongpingensis* and *P. mume* but absent in the apricot group as candidate SVs (Fig. 5B). As a result, we found 117 deletion SVs and then extracted the genes within a distance of ~2000­ bp from SVs. The functional annotation of 177 SV-associated genes showed that they were related to plant growth and development and environmental adaptability (Supplementary Data Table S6).

After that, we focused on genes associated with low temperatures. Notably, we found that a 566-bp deletion (SV) was present in *P*. *zhengheensis, P. mume* and *P. hongpingensis*, but absent in *P. armeniaca* (Fig. 5C). In other words, there was an insertion (SV) of 566 bp in apricot, which was not present in *P*. *zhengheensis*, *P. mume* or *P. hongpingensis*. This insertion was positioned 1.6 kb upstream of *HSFA1d* (the gene name is PARG23073, originating from apricot CZH, and located on Chr 7.) and encoded a heat shock factor protein that is probably responsible for plant growth during chilling [17]. The promoter sequences of *PzHSFA1d* and *PaHSFA1d* were cloned to 1626 and 2223 bp, respectively (Supplementary Data Fig. S4). Then, the Plant Cis-Acting Regulatory Element (PlantCARE) database was used to analyse the *cis*-acting elements. *PaHSFA1d* had more *cis*-acting element types than *P*. *zhengheensis*, e.g. GTGGC-motif, MYB-like sequence, CGTCA-motif, P-box, GT1-motif, AT-rich sequence, as-1, TGACG-motif (Supplementary Data Fig. S5), LTRE, WUN-motif, ERE, DRE core, I-box, STRE, TCT-motif, TATA, TC-rich repeats and WRE3. Of these *cis*-acting elements, the last 10 existed in the 566-bp SV fragments (Fig. 5C). The DRE core responded to drought and low-temperature stress, and the LTRE element was involved in low-temperature responsiveness [29, 30]. The STRE element could be activated by heat shock, osmotic stress, low pH and nutrient starvation [31]. Therefore, we speculated that the promoter insertion of *HSFA1d* in apricot enhances temperature adaptation to the extent that its main distribution is in relatively colder regions of China.

### *HSFA1d* regulates apricot temperature adaptability

To confirm our conjecture, we collected winter branches from *P*. *zhengheensis*, *P. mume* and *P. armeniaca*, and subsequently measured the expression levels of the *HSFA1d* gene. The relative RNA expression level of the *HSFA1d* gene in *P. armeniaca* was significantly higher than that in *P. zhengheensis* and *P. mume* during a longer period of low temperature (Supplementary Data Fig. S6). Due to the remarkable protein sequence similarity of 99.9% between *P. zhengheensis* and *P. mume*, *P*. *zhengheensis* and *P. armeniaca* were used as research materials for further experiments (Supplementary Data Fig. S7). The expression of the *HSFA1d* gene in *P*. *zhengheensis* and apricot branches was examined at a low temperature (4°C). The relative RNA expression level of the *HSFA1d* gene in apricot was significantly higher than that in *P*. *zhengheensis* after 4°C treatment for 12 h, while there was no significant difference in the expression level under normal temperature treatment (25°C) (Fig. 6A).

We then cloned the CDSs of *PzHSFA1d* (*P*. *zhengheensis*) and *PaHSFA1d* (apricot) (Supplementary Data Fig. S8). Multiple alignment of the amino acid sequences showed that *PzHSFA1d* and *PaHSFA1d* had 98% sequence similarity and identical conserved domains. To verify the gene function of *HSFA1d* in the two species, we heterologously transformed *PaHSFA1d* and *PzHSFA1d* in *A. thaliana*. The hypocotyl length of overexpressing *A. thaliana* (OE) treated at 4°C was significantly longer than that of the wild-type (WT), whereas there was no significant difference in hypocotyl length between OE and WT plants treated at 25°C. In addition, at 4°C the cell length of OE *A. thaliana* was significantly longer than that of WT plants, while there was no significant difference in cell length between OE and WT plants at 25°C (Fig. 6B). Additionally, we subjected transgenic *A. thaliana* to cold treatment, and the results revealed that transgenic *A. thaliana* exhibited greater resistance to low temperatures compared with WT (Supplementary Data Figs S9 and S10). These results suggest that *PzHSFA1d* and *PaHSFA1d* may positively regulate plant growth in *P*. *zhengheensis* and apricot under chilling.

Because *PzHSFA1d* and *PaHSFA1d* had the same gene function, we hypothesized that their differential expression was related to promoter insertion. Therefore, we used a dual luciferase assay to confirm the impact of SV insertion on promoter activity. The promoters of *PzHSFA1d* and *PaHSFA1d* were individually fused with the luciferase reporter gene (LUC) in the pGreenII-0800-LUC vector. The resulting vectors, pro*PzHSFA1d*::LUC and pro*PzHSFA1d*::LUC, were transformed into *Agrobacterium tumefaciens* GV3101 (pSoup). The relative luciferase activity of pro*PaHSFA1d* was significantly higher than that of pro*PzHSFA1d* at low temperatures (Fig. 6C). The results indicate that insertion in the promoter region of the *PaHSFA1d* gene confers a stronger active ability than that in *PzHSFA1d*. Thus, this SV in the promoter may have led to increased expression of *HSFA1d*, consequently contributing to temperature adaptation in apricot.

## Discussion

The *Armeniaca* group includes 10 species, the most famous of which are *P. mume* and apricot [67]. Although Zhang identified *P*. *zhengheensis* as a new species of the *Armeniaca* section in 1996, other researchers have suggested different insights. Wang *et al*. [68] supported *P*. *zhengheensis* as a variety of *P. mume*. The phylogenetic tree analysis based on the ITS sequence by Chen *et al*. [6] supported the classification of *P*. *zhengheensis* as a variety of *P. mume*. Through the molecular phylogenetic study and DNA barcode exploration of the major fruit tree germplasm resources of the genus *Prunus*, Zhang *et al*. [7] found that *P*. *zhengheensis* and *P. mume* were classified into a group based on chloroplast SSR marker analysis. Huang *et al*. [8] confirmed that *P*. *zhengheensis* was closely related to *P. mume* using the complete chloroplast genome.

In our study, the phylogenetic tree based on nuclear single-copy genes revealed that *P*. *zhengheensis* was more closely related to apricot, while a phylogenetic tree based on the organelle genome revealed that *P*. *zhengheensis* was more closely related to *P. mume*. The phylogenetic incongruence between nuclear and organelle genomes can have several causes, including episodes of convergent evolution, ILS, and introgression hybridization. Hybridization and convergent evolution occur after species formation, but ILS occurs during species formation, particularly in the rapid succession of speciation events [69]. ILS has been described in many phylogenetic clades, including primates [70], marsupials [27] and bryophytes [71]. QuIBL is a tree-based method that uses internal branch length to distinguish between introgression and ILS. It has been applied to several species, including *Eospalax* [72], *Catalpa* [27], *Triplophysa* [73], bryophytes [71], *Echinochloa* spp. [74] and *Gossypium* [75]. In our study, the results of QuIBL showed that ILS was the main reason for the contradictory classification.

LTR retrotransposons, such as Ty1/Copia and Ty3/Gypsy retrotransposons, are enriched in pericentromeric regions, playing important roles in maintaining chromatin structures and centromere functions, and are suggested to be significant drivers of genome evolution, gene expression, and adaptation to environmental changes among plant lineages [76–78]. Papolu *et al*. [79] concluded that LTRs could regulate *PHRE1* and *PHRE2* activity in moso bamboo under heat stress. Sun *et al*. [80] found that LTR retrotransposons indicated that recent repeat amplification may contribute to adaptive evolution and species differentiation of the desert poplar genome. In our study, the analysis of insertion times reveals that five Armenian species have experienced bursts of different LTR types over the past 3.5 million years. In particular, at 0.25 and 0.5 MYA, LTR–RT bursts peaked. During this period, the Earth was experiencing climatic oscillations known as the Pleistocene Epoch, and it is possible that the insertion of LTRs in the genome affected the expression of resistance genes, thereby enhancing the plant's adaptability to the environment. Nevertheless, more thorough and in-depth research is needed to understand the underlying process causing the LTR burst.

Apricot is a well-known fruit tree around the world. Its blooms can be used as ornaments, and the fruit is juicy and can be eaten raw or preserved. The kernel can also be consumed. Our findings indicate that *P*. *zhengheensis* is more closely related to apricot than *P. mume*, although it originated in South China and apricot is mainly distributed in the cold north. As a result, *P*. *zhengheensis* is an excellent research material for investigating the mechanisms underlying apricot cold resistance. In this study, we developed a comprehensive SV map of the *Armeniaca* section (Fig. 5B), which enabled us to investigate the potential mechanisms influencing nearby genes. We classified SVs based on the geographical distribution characteristics of the *Armeniaca* section, focusing on the SVs present in *P*. *zhengheensis* and *P. mume* but absent in apricot. Finally, an insertion SV in the promoter of the *HSFA1d* gene in apricot that was absent in *P*. *zhengheensis* was identified. *HSFA1d* is a heat shock protein, and it has been reported that this gene can respond to both high and low temperatures [25]. Therefore, we hypothesized that the promoter insertion of the *HSFA1d* gene into apricot enhanced the expression of the *HSFA1d* gene, thereby improving the low-temperature adaptability of apricot.

To verify our speculation, we first verified that the expression level of the *HSFA1d* gene in apricot treated at a low temperature was significantly higher than that in *P*. *zhengheensis*, indicating that the *HSFA1d* gene is the cause of cold tolerance in apricot. Next, we verified the function of the *HSFA1d* gene in *P*. *zhengheensis* (*PzHSFA1d*) and apricot (*PaHSFA1d*). The overexpression of *PzHSFA1d* and *PaHSFA1d* in *A. thaliana* showed that *HSFA1d* from both species could maintain plant growth at low temperature. The dual luciferase assay confirmed that the SV-inserted promoter had stronger activity at low temperatures. These findings revealed that SV insertion was responsible for the different expression levels of *PaHSFA1d* and *PzHSFA1d*, possibly related to the low-temperature adaptation of apricots. It is not rare for promoter variation to influence gene expression and consequently affect plant phenotype or resistance. For example, the insertion of 716 bp in the promoter of *PMA5G03691*.*1* affected the responsiveness of nearby genes to heat, potentially leading to pearl millet seed grown at higher temperatures [13]. Lin *et al*. discovered sequence mutations in the promoter regions of two key genes that may play a significant role in *F*. *tartaricum*’s high rutin content and self-reproduction [81]. In addition, a natural promoter variation of *SlBBX31* improved cold tolerance during tomato domestication [82].

HSFs are highly conserved key regulators of the heat stress response in mammals, yeast and plants [83]. Among these HSF factors, the master regulators of the heat shock response in *A. thaliana* are *HSFA1a*, *HSFA1b* and *HSFA1d*. At present, the majority of investigations on the *HSFA1* gene are concerned with heat. For example, Tan *et al*. reported that under warm daytime conditions, *HSFA1* accumulated significantly and entered the nucleus, interacted with PIF4, and stabilized PIF4 by interfering with the photochrome B-PIF4 interaction [83]. In addition to responding to high temperatures, *HSFA1* also responded to low temperatures. Under cold conditions, *NPR1* oligomers in the cytoplasm produce monomers that translocate to the nucleus and interact with *HSFA1* to induce *HSFA1*-regulated gene induction and cold adaptation [84]. Qi *et al*. revealed that a heat-shock pretreatment improves cucumber seedling tolerance to cold stress and that *CsHSFA1d* and jasmonic acid play essential roles in this cold acclimation process [25]. In addition, by regulating the expression of the ribosomal protein genes (*RPL9* and *RPL18*), *HSFA1d* facilitated hypocotyl elongation under chilling and sustained protein translation for growth [17]. Our study also showed that the *HSFA1d* gene can positively regulate low-temperature adaptation. However, the mechanism underlying apricot's cold tolerance is multifaceted. Although our study gives some evidence for apricot's adaptation to low temperatures, further research is needed in the future.

Our study has established a foundational framework for investigating apricot's adaptability to low temperatures. Furthermore, it has contributed to a deeper comprehension of apricot distribution mechanisms, providing crucial data support for the breeding of apricot group fruit trees.

### Conclusions

In this study, we report the first haplotype-resolved chromosome-level genome of *P*. *zhengheensis* (hap1 and hap2). Based on this high-quality genome, ILS has been indicated to contribute to the conflicting phylogenetic relationships between nuclear and organelle phylogenies. Together with the *P*. *zhengheensis* genome, we built a graph-based pan-genome and performed SV calling, enabling the identification of variants potentially associated with apricot cold adaptation. A natural 566-bp insertion SV was identified in the promoter region of the *HSFA1d* gene of apricot, which might clarify why apricot was adaptive in northern China, while *P*. *zhengheensis* and *P. mume* were distributed in southern China.

## Materials and methods

### Sample preparation and sequencing

Fresh samples of plants were collected from Zhenghe County, Fujian, China (27°18′24″ N, 118°59′48″ E and 930 m above sea level). Considering that *P*. *zhengheensis* is an endangered species in China, we used fresh shoots for plant tissue culture and subsequent experiments to conserve the species. The genomic DNA was extracted to construct 150-bp paired-end libraries and then sequenced on the Illumina HiSeq X Ten platform. For CCS, the procedures were performed according to the standard protocol provided by PacBio, including sample quality testing, library construction, library quality testing and library sequencing. For Hi-C sequencing, the library was prepared using standard protocols and sequenced on the Illumina HiSeq X Ten platform. In addition, plant roots, stems and leaves were collected for long-read ONT transcriptome sequencing.

### Genome assembly

The clean Illumina data were analysed using Jellyfish v2.2.10 [32] software for the genome survey, and the results were visualized using GenomeScope 2.0 [33]. The raw genomic HiFi reads were converted to highly accurate single-molecule CCS reads. Hifiasm v0.16.1-r375 software [34] was used to assemble a pair of haplotype-resolved contigs (hap1 and hap2) based on CCS reads and Hi-C data. The assembly results were then corrected using Plion v1.24 [35], and Purge_dups v1.2.5 [36] software was used for redundancy removal. Subsequently, the two sets of haplotype genomes were chromosome-mounted into eight chromosomes using Juicer v1.6 [37] and the 3D-DNA v190716 pipeline (https://github.com/theaidenlab/3d-dna) based on Hi-C data. Finally, the results were visualized and corrected manually using Juicebox Assembly Tools software [38].

### Genome annotation

The repeat sequence database was constructed using RepeatModeler v2.0.2 [39] and repetitive sequences were identified using RepeatMasker (open −4.07) [40]. Protein-coding genes were annotated using the BRAKER v2.1.6 [41] genome annotation pipeline, which integrates both *ab initio* gene predictions generated by Augustus v3.4.0 [42] and GeneMark-ET [43] and full-length transcriptome data, as well as protein sequences for five related *Prunus* species (*P. persica*, *P. mume*, *P*. *mira*, *P. armeniaca*, *P. salicina*, *P. avium*). The gene annotation integrity was evaluated using BUSCO software (with the parameters —lineage_dataset: embryophyta_odb10).

### Phylogeny based on the nuclear genome

*Prunus zhengheensis* and another 24 genomes were used to reconstruct the phylogeny, including *P. mume* [44], *P. mume* var. *tortuosa* [45], six cultivars of apricot [*P. armeniaca* ‘Chuanzhihong’ [46], *P. armeniaca* ‘Yinxiangbai’ [47], *P. armeniaca* ‘Longwangmao’ (https://www.rosaceae.org/Analysis/10254126), *P. armeniaca* ‘Jintaiyang’ (https://www.rosaceae.org/Analysis/10254125), *P. armeniaca* ‘Stella’, *P. armeniaca* accession ‘Marouch #14’], *P*. *sibirica* and *P*. *mandshurica* [1], *P. hongpingensis* [16], *P. yedoensis* var. *nudiflora* [48]*, P. serrulata* [49], *P. avium* [50], *P. persica* [51], *P*. *misa* [52], *P. salicina* ‘Zhongli No. 6’ [53], *Pyrus communis* [54], *Rubus occidentalis* [55], *Malus* × *domestica* [56], *Fragaria vesca* [57] and *Vitis vinifera* [58].

For phylogenetic analysis, we used two different approaches: the concatenation-based method (maximum likelihood) and the coalescence-based approach (ASTRAL). OrthoFinder v2.4.0 [59] was used for gene family clustering in 23 species, and 155 single-copy orthologue sequences were used to construct a phylogenetic tree based on the best model JTT + I + G4 + F with 1000 bootstraps using IQ-TREE v2.1.2 [60]. For coalescent inference, gene trees were generated for all single-copy sequences, and species trees were inferred using ASTRAL v5.7.8 [61] based on all single-copy gene trees.

### Phylogenetic analysis of *P*. *zhengheensis* organelle genomes

According to previous studies [1, 47, 62], we downloaded *P. mume*, *P. hongpingensis* and apricot resequencing data (Supplementary Data Table S7), and Getorganelle software was used to assemble the chloroplast genomes of *P*. *zhengheensis*, *P. armeniaca*, *P*. *sibirica* and *P*. *mandshurica*. DnaSP v6 software was employed to calculate the chloroplast haplotype, and PopART was used to depict the chloroplast haplotype network.

For the mitochondrial genome, the *P*. *zhengheensis* mitochondrial genome was assembled using nextDenovo v2.5.0 software and annotated using MitoFinder v1.4. In addition, we assembled the mitochondrial genome of *P. hongpingensis* using Getorganelle software. A total of 32 shared protein sequences from the mitochondrial genomes of *P*. *zhengheensis* and other *Prunus* species (Supplementary Data Table S8) were extracted for the construction of phylogenetic trees using IQ-TREE v2.1.2 based on the JTTDCMut + F + R3 model.

### QuIBL analysis based on whole-genome alignment

RepeatMasker version 4.1.0 was used to mask genomic repetitive sequences, and Cactus v2.4.4 [63] was used for multi-species whole-genome alignment. After obtaining the hal file, hal2maf [64] was used to convert hal into the maf format, and mafSplit was used to split the hal by chromosome. msa_view was used to transfer maf to fasta, and msa_view — aggregate was used to combine the split chromosomes to obtain a mutimatrix. To reduce the likelihood of sampling a window containing a recombination breakpoint, we extracted 1-k windows separated by 20 kb from the muti-matrix using a Seqkit. A window of <10 simple information sites was filtered out. IQ-TREE v2.1.2 was used to construct a phylogenetic tree, and the resulting phylogenetic tree was analysed for ILS and introgression using QuIBL software [65] (apple was the outgroup).

### Pan-genome of *Armeniaca* species

Eleven *Armeniaca* genomes, namely *P*. *zhengheensis*, *P. mume*, *P. mume* var. *tortuosa*, six cultivars of apricot (*P. armeniaca* ‘Chuanzhihong’, *P. armeniaca* ‘Yinxiangbai’, *P. armeniaca* ‘Longwangmao’, *P. armeniaca* ‘Jintaiyang’, *P. armeniaca* ‘Stella’, *P. armeniaca* accession ‘Marouch #14’), *P*. *sibirica* and *P*. *mandshurica*, were used for the family-based pan-genome. The present study established core, softcore, dispensable and private gene families within the *Armeniaca* section through a comparative genomics approach based on Orthofinder v2.4.0 software. Core gene families were shared by 13 genomes, and softcore gene families were shared by 10 genomes. Dispensable gene families were shared by two to nine Armenian species. Furthermore, the presence of specific gene families in a single genome was determined to be private. psvcp_v1.01 [66] software was used to construct the graph-based pan-genome, and *P*. *zhengheensis* was the primary reference.

### Structural variation identification

To identify SVs, all 13 genomes were realigned to the pan-genome using MUMmer v4.00, and SVs were derived from alignments using Assemblytics v1.2.1.

### qRT–PCR and generation of transgenic plants

After the plant materials had been treated at room temperature (25°C) and low temperature (4°C) for 0, 2, 6, 8 and 12 h, total RNA was extracted using the Tiangen RNA Extraction Kit (Beijing, China), and qRT–PCR was performed to determine the gene expression level. Subsequently, we cloned the CDS of the *HSFA1d* gene of *P*. *zhengheensis* and apricot, and constructed sequences inserted into the carrier plasmid of pCAMBIA2301.

The constructed carrier was transformed into *A. tumefaciens* GV3101 and cultured at 28°C for 2–3 days. Colonies were selected for PCR identification. The identified colonies were placed in LB medium containing kanamycin and rifampicin at 28°C and cultured to an OD600 value of 0.6–0.8. *Arabidopsis thaliana* was infected with *Agrobacterium* using the floral dip method and cultured in the dark for 12 h under normal light. After the seeds had been collected, they were disinfected and placed on MS medium containing kanamycin, and the plants that grew normally were transplanted into a greenhouse for culture. PCR identification and GUS staining were then used to screen the positive plants. Seeds produced by the positive plants were disinfected, placed in MS medium, purified at 4°C for 3 days and exposed to light for 2 days. Fully germinated transgenic and WT seedlings were transferred to new MS medium and cultured in the dark for 3 days to observe the phenotypes. Three strains of *P*. *zhengheensis* and apricot were selected for qRT–PCR analysis. The hypocotyl length was measured, and the hypocotyl cells were observed using a microscope.

### Transient luciferase expression assays

The promoters of *PzHSFA1d* and *PaHSFA1d* were individually fused with the luciferase reporter gene (LUC) in the pGreenII-0800-LUC vector. The resulting vectors, pro*PzHSFA1d*::LUC and pro*PaHSFA1d*::LUC, were transformed into *A. tumefaciens* GV3101 (pSoup) and cultured at 28°C for 3 days. Normally growing colonies were selected for PCR identification, and the identified colonies were cultured overnight in LB cultures containing kanamycin and rifampicin. Solutions of 10 mM MES, 10 mM MgCl_2_ and 100 mM acetosyringone were added for resuspension, and the culture was diluted to an OD600 of 0.6. Tobacco leaves were then injected with the culture, cultivated in the dark for 2 days, and then cultured at 4°C and 25°C for 12 h each. All primers used in this study are shown in Supplementary Data Table S9.

## Supplementary Material

Web_Material_uhae103

## Data Availability

The assembled genome sequences of *P*. *zhengheensis* were deposited in the Genome Database for Rosaceae (https://www.rosaceae.org/).
