# Supplementary material for: Haplotype-resolved genome of Prunus zhengheensis provides insight into its evolution and low temperature adaptation in apricot
Source: Hortic Res. 2024 Apr 8;11(4):uhae103. doi: 10.1093/hr/uhae103 (PMC11059810; doi:10.1093/hr/uhae103)
Supplement: Web_Material_uhae103 [file web_material_uhae103.zip › Fig. S7. Comparative analysis of PzHSFA1d and PmHSFA1d protein sequences.pdf]

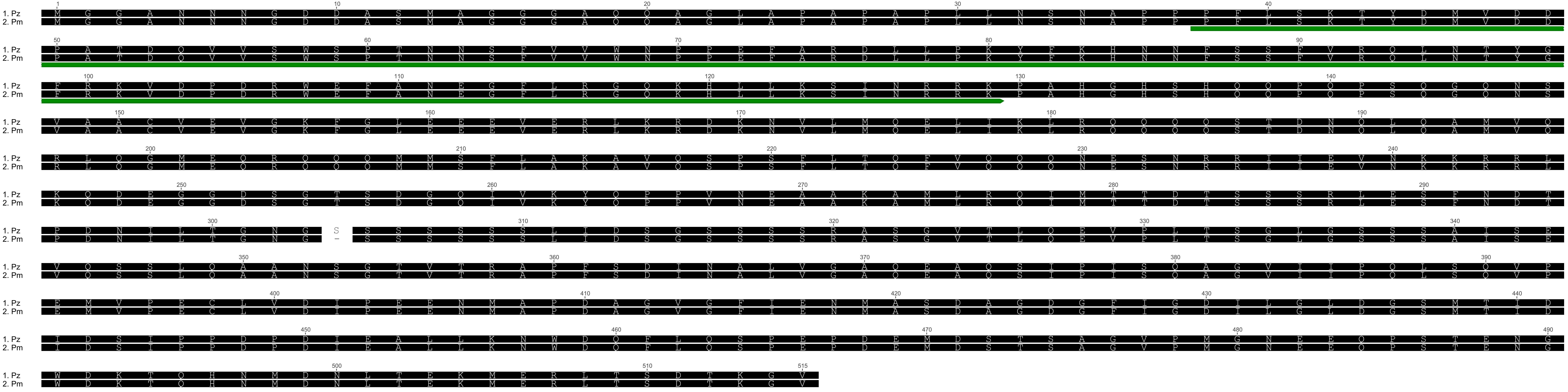
1. Pz MGGANNNGDDASMAGGGAQQAGLAPAPAPLLNSNAPPPFLSKTYDMVDD
2. Pm MGGANNNGDDASMAGGGAQQAGLAPAPAPLLNSNAPPPFLSKTYDMVDD
1. Pz PATDQVVSWSPTNNSFVVWNPPEFARDLLPKYFKHNNFSSFVRQLNTYG
2. Pm PATDQVVSWSPTNNSFVVWNPPEFARDLLPKYFKHNNFSSFVRQLNTYG
1. Pz FRKVDPDRWEFANEGFLRGQKHLLKSINRRKPAHGHSHQQPQPSQGQNS
2. Pm FRKVDPDRWEFANEGFLRGQKHLLKSINRRKPAHGHSHQQPQPSQGQNS
1. Pz VAACVEVGKFGLEEEVERLKRDKNVLMQELIKLRQQQQSTDNQLQAMVQ
2. Pm VAACVEVGKFGLEEEVERLKRDKNVLMQELIKLRQQQQSTDNQLQAMVQ
1. Pz RLQGMEQRQQQMMSFLAKAVQSPSFLTQFVQQQNESNRRIIEVNKKRRL
2. Pm RLQGMEQRQQQMMSFLAKAVQSPSFLTQFVQQQNESNRRIIEVNKKRRL
1. Pz KQDEGGDSGTSDGQIVKYQPPVNEAAKAMLRQIMTTDTSSSRLESFNDT
2. Pm KQDEGGDSGTSDGQIVKYQPPVNEAAKAMLRQIMTTDTSSSRLESFNDT
1. Pz PDNILTGNGSSSSSSSLIDSGSSSSRASGVTLQEVPLTSGLGSSSAISE
2. Pm PDNILTGNG-SSSSSSLIDSGSSSSRASGVTLQEVPLTSGLGSSSAISE
1. Pz VQSSLQAANSGTVTRAPFSDINALVGAQEAQSIPISQAGVIIPQLSQVP
2. Pm VQSSLQAANSGTVTRAPFSDINALVGAQEAQSIPISQAGVIIPQLSQVP
1. Pz EMVPECLVDIPEENMAPDAGVGFIENMASDAGDGFIGDILGLDGSMTID
2. Pm EMVPECLVDIPEENMAPDAGVGFIENMASDAGDGFIGDILGLDGSMTID
1. Pz IDSIPPDPDIEALLKNWDQFLQSPEPDEMDSTSAGVPMGNEEQPSTENG
2. Pm IDSIPPDPDIEALLKNWDQFLQSPEPDEMDSTSAGVPMGNEEQPSTENG
1. Pz WDKTQHNMDNLTEKMERLTSDTKGV
2. Pm WDKTQHNMDNLTEKMERLTSDTKGV
1 10 20 30 40 50 60 70 80 90 100 110 120 130 140 150 160 170 180 190 200 210 220 230 240 250 260 270 280 290 300 310 320 330 340 350 360 370 380 390 400 410 420 430 440 450 460 470 480 490 500 510 515
