# Supplementary material for: Haplotype-resolved genome of Prunus zhengheensis provides insight into its evolution and low temperature adaptation in apricot
Source: Hortic Res. 2024 Apr 8;11(4):uhae103. doi: 10.1093/hr/uhae103 (PMC11059810; doi:10.1093/hr/uhae103)
Supplement: Web_Material_uhae103 [file web_material_uhae103.zip › Fig. S8. Comparison of HSFA1d coding sequences of P. zhengheensis and apricot.pdf]

```
           1       10        20        30        40        50        60        70        80        90        100       110       120       130
           |-------+---------+---------+---------+---------+---------+---------+---------+---------+---------+---------+---------+---------|
PzHSFA1d   MGGANNNGDDASMAGGG---AQQAGLAPAPAPLLNSNAPPPFLSKTYDMVDDPATDQVVSWSPTNNSFVVWNPPEFARDLLPKYFKHNNFSSFVRQLNTYGFRKVDPDRWEFANEGFLRGQKHLLKSINR
PaHSFA1d   MGGANNNGDDASMAGGGGGGAQQAGLAPAPAPLLNSNAPPPFLSKTYDMVDDPATDQVVSWSPTNNSFVVWNPPEFARDLLPKYFKHNNFSSFVRQLNTYGFRKVDPDRWEFANEGFLRGQKHLLKSINR
Consensus  MGGANNNGDDASMAGGG...AQQAGLAPAPAPLLNSNAPPPFLSKTYDMVDDPATDQVVSWSPTNNSFVVWNPPEFARDLLPKYFKHNNFSSFVRQLNTYGFRKVDPDRWEFANEGFLRGQKHLLKSINR

           131     140       150       160       170       180       190       200       210       220       230       240       250       260
           |-------+---------+---------+---------+---------+---------+---------+---------+---------+---------+---------+---------+---------|
PzHSFA1d   RKPAHGHSHQQPQPSQGQNSVAACVEVGKFGLEEEVERLKRDKNVLMQELIKLRQQQQSTDNQLQAMVQRLQGMEQRQQQMMSFLAKAVQSPSFLTQFVQQQNESNRRIIEVNKKRRLKQDEGGDSGTSD
PaHSFA1d   RKPAHGHSHQQPQPSQGQNSVAACVEVGKFGLEEEVERLKRDKNVLMQELIKLRQQQQSTDNQLQAMVQRLQGMEQRQQQMMSFLAKAVQSPSFLTQFVQQQNESNRRIIEVNKKRRLKQDEGGDSGTPD
Consensus  RKPAHGHSHQQPQPSQGQNSVAACVEVGKFGLEEEVERLKRDKNVLMQELIKLRQQQQSTDNQLQAMVQRLQGMEQRQQQMMSFLAKAVQSPSFLTQFVQQQNESNRRIIEVNKKRRLKQDEGGDSGTpD

           261     270       280       290       300       310       320       330       340       350       360       370       380       390
           |-------+---------+---------+---------+---------+---------+---------+---------+---------+---------+---------+---------+---------|
PzHSFA1d   GQIVKYQPPVNEAAKAMLRQIMTTDTSSSRLESFNDTPDNILTGNGSSSSSSSLIDSGSSSSRASGVTLQEVPLTSGLGSSSAISEVQSSLQAANSGTVTRAPFSDINALVGAQEAQSIPISQAGVIIPQ
PaHSFA1d   GQIVKYQPPVNEAAKAMLRQIMTTDTSSSRLESFNDTPDNILTGNGSSSSSS-LIDSGSSSSRASGVTLQEVPLTSGLGSSSAISEVQSSLQAANSGTVTRAPFSDINALVGAQEAQSIPISQAGVIIPE
Consensus  GQIVKYQPPVNEAAKAMLRQIMTTDTSSSRLESFNDTPDNILTGNGSSSSSS.LIDSGSSSSRASGVTLQEVPLTSGLGSSSAISEVQSSLQAANSGTVTRAPFSDINALVGAQEAQSIPISQAGVIIP#

           391     400       410       420       430       440       450       460       470       480       490       500       510     518
           |-------+---------+---------+---------+---------+---------+---------+---------+---------+---------+---------+---------+-------|
PzHSFA1d   LSQVPEMVPECLVDIPEENMAPDAGVGFIENMASDAGDGFIGDILGLDGSMTIDIDSIPPDPDIEALLKNWDQFLQSPEPDEMDSTSAGVPMGNEEQPSTENGWDKTQHNMDNLTEKMERLTSDTKGV
PaHSFA1d   LSQIPEMVPECLVDIPEENMAPDAGVGFIENMASDAGDGFIGDILGLDGSMTIDIDSIPPDPDIEALLKNWDQFLQSPEPDEMDSTSAGVPMGNEEQPSTENGWDKTQHNMDNLTEKMERLTSDTKGV
Consensus  LSQ!PEMVPECLVDIPEENMAPDAGVGFIENMASDAGDGFIGDILGLDGSMTIDIDSIPPDPDIEALLKNWDQFLQSPEPDEMDSTSAGVPMGNEEQPSTENGWDKTQHNMDNLTEKMERLTSDTKGV
```
