# Supplementary material for: Haplotype-resolved genome of Prunus zhengheensis provides insight into its evolution and low temperature adaptation in apricot
Source: Hortic Res. 2024 Apr 8;11(4):uhae103. doi: 10.1093/hr/uhae103 (PMC11059810; doi:10.1093/hr/uhae103)
Supplement: Web_Material_uhae103 [file web_material_uhae103.zip › Supplemental Figures.docx]

**Supplementary files**

**Supplementary Figure**


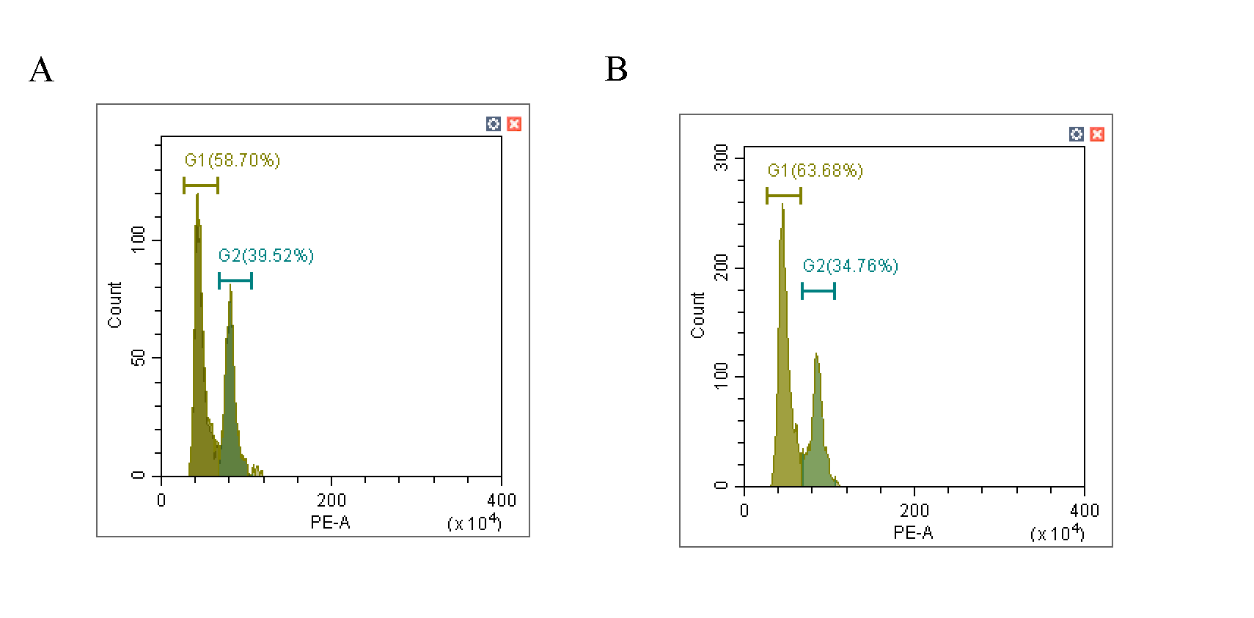


**Fig. S1. The genome ploidy analysis of *P. zhengheensis* by using** **flow cytometry.** (A) The diploid *P. mume* was used as a control. (B) The flow cytometry result of *P. zhengheensis*. The x axis is the fluorescence intensity of PI from a single cell. The y axis indicates the number of cells collected by flow.


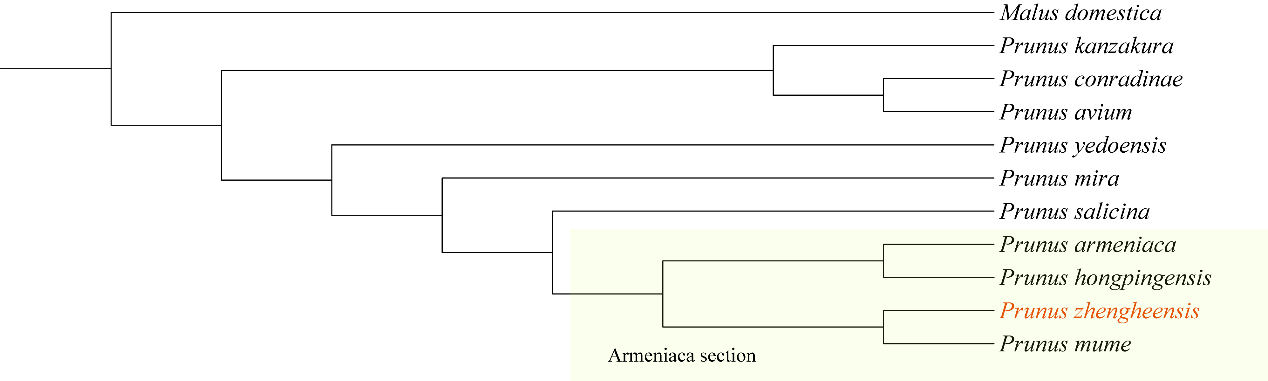


**Fig. S2. Phylogenetic reconstruction of *P. zhengheensis* and other *Prunus* species based on mitochondrial genomes.**


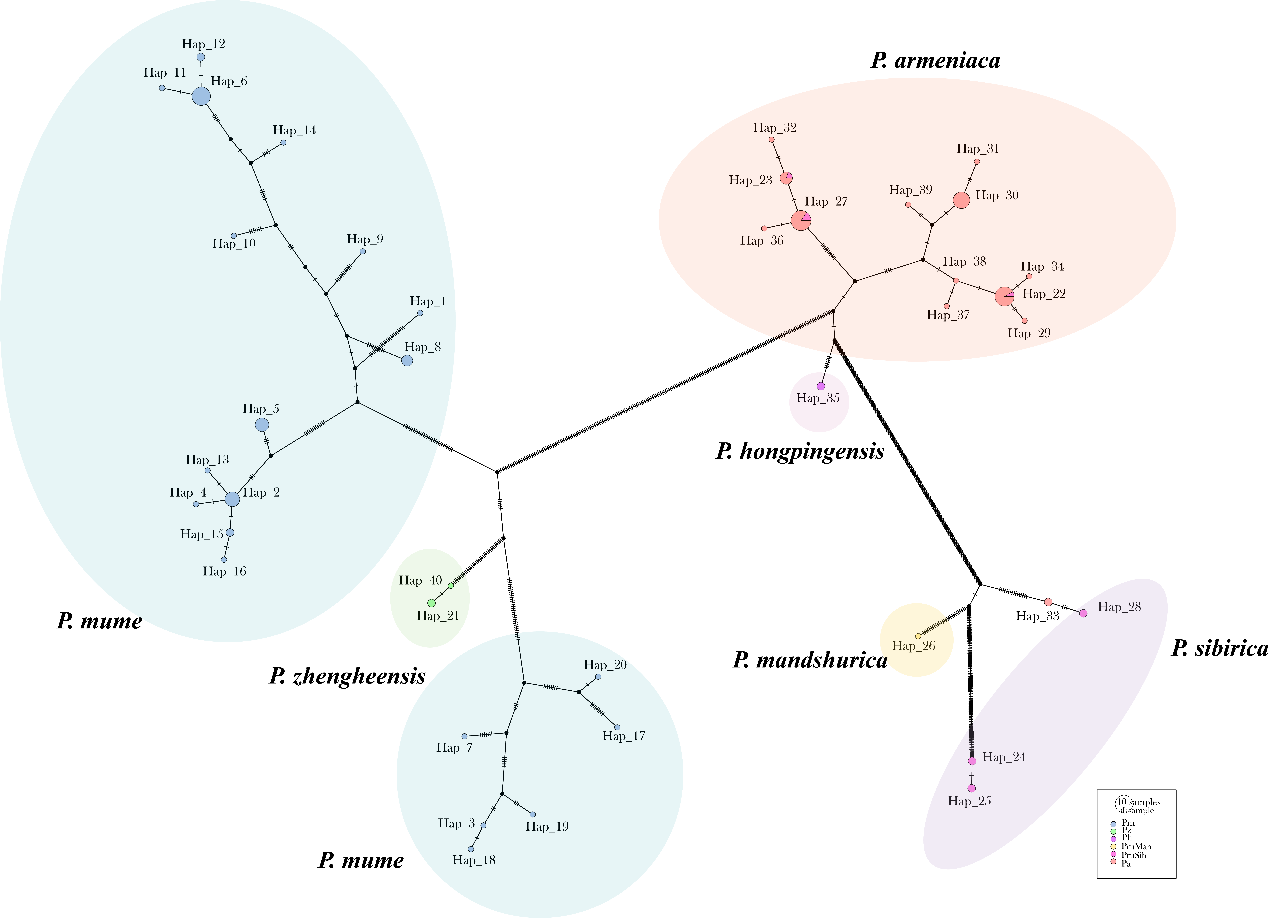


**Fig. S3. The genetic relationships among Armeniaca chloroplastic haplotypes are depicted by a median-joining network.** The size of the circles indicates sample size. The blue circle represents *P. mume*, the green represents *P. zhengheensis*, the orange represents apricot, the yellow represents *P. mandshurica*, the purple represents *P. sibirica* and the lilac represents *P. hongpingensis.*


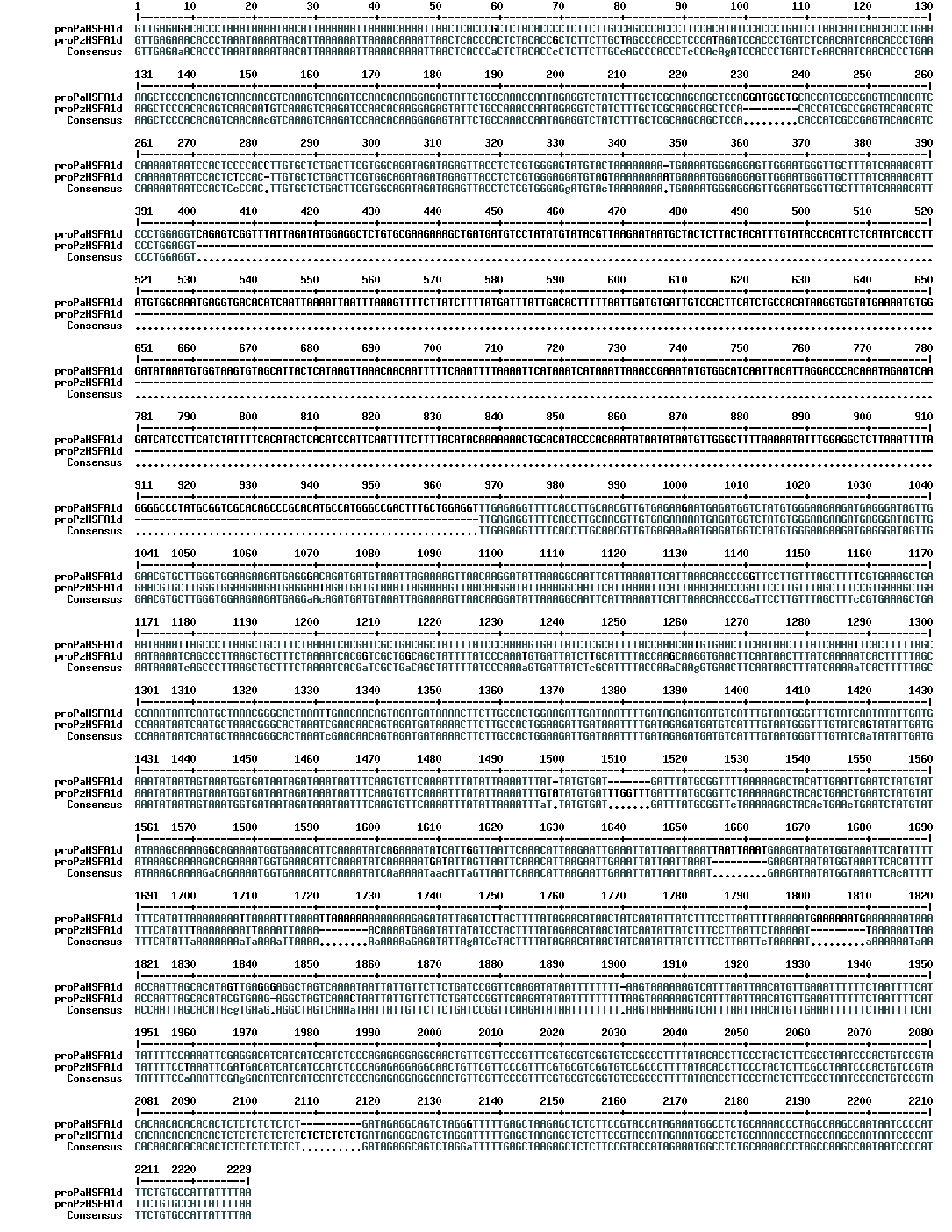


**Fig. S4. The sequence comparison between *PaHSFA1d* and *PzHSFA1d* promoters.**


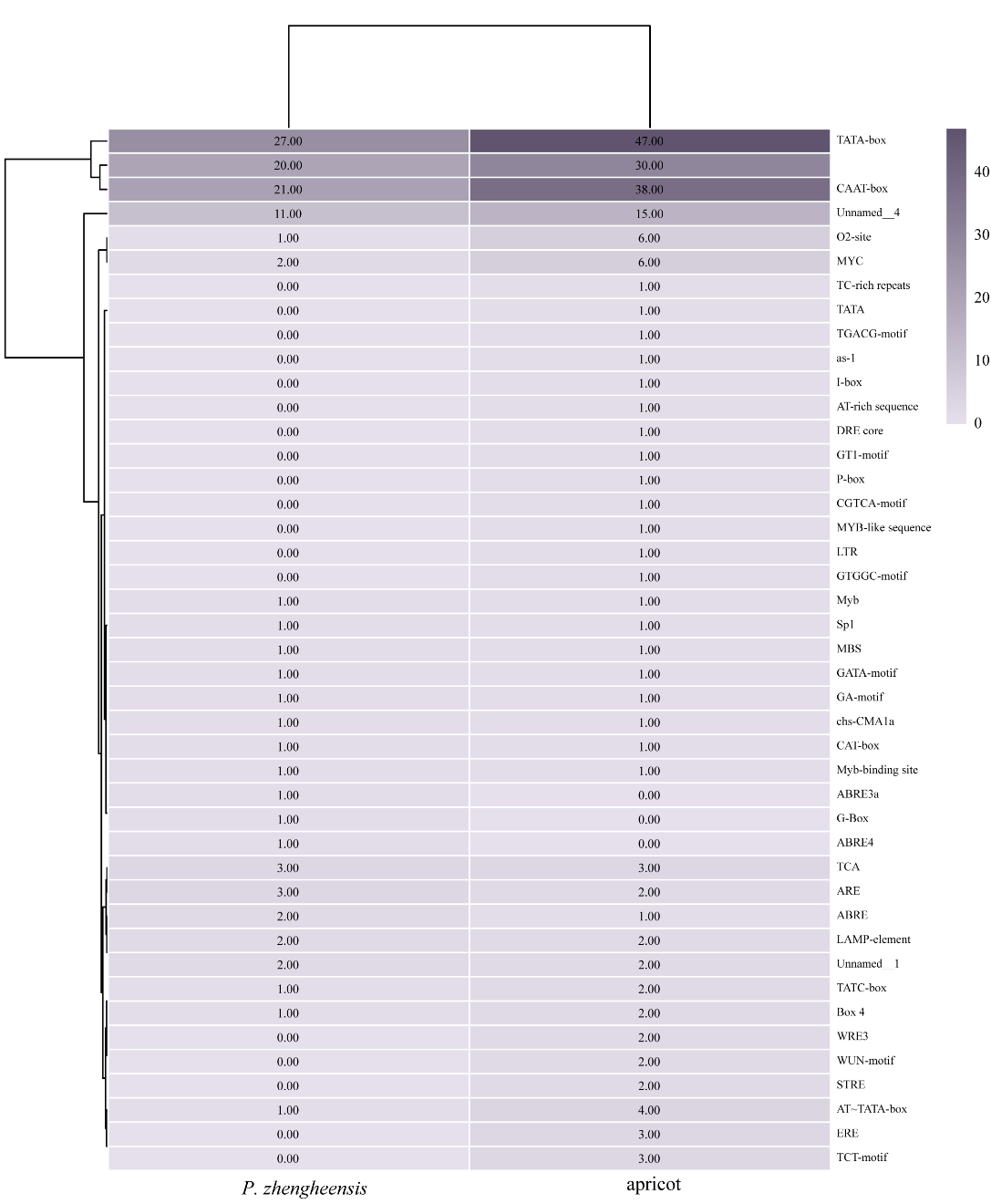


**Fig. S5. Promoter cis-acting element analysis.**


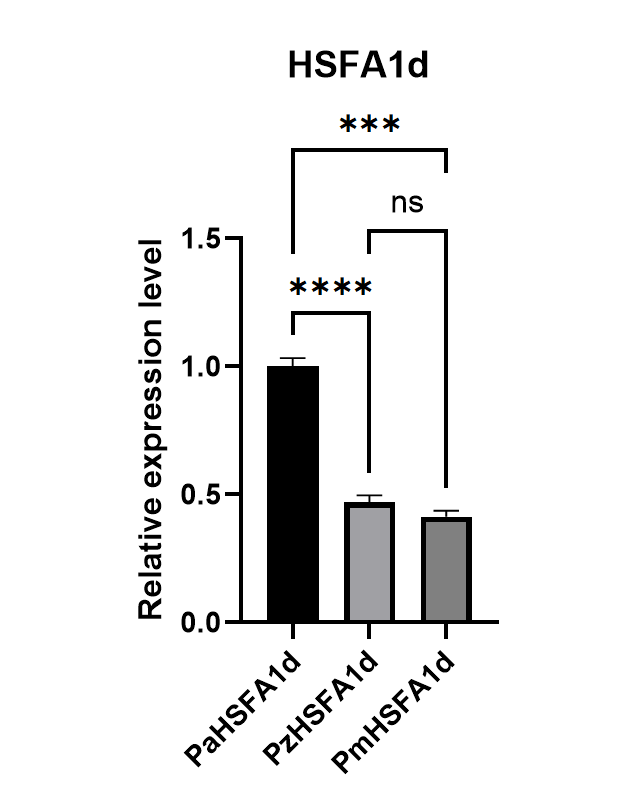
­­

**Fig. S6. The expression levels of *HSFA1d* (*PzHSFA1d*,** ***PaHSFA1d* and *PmHSFA1d*) gene were confirmed in winter branches by RT-qPCR analysis.**


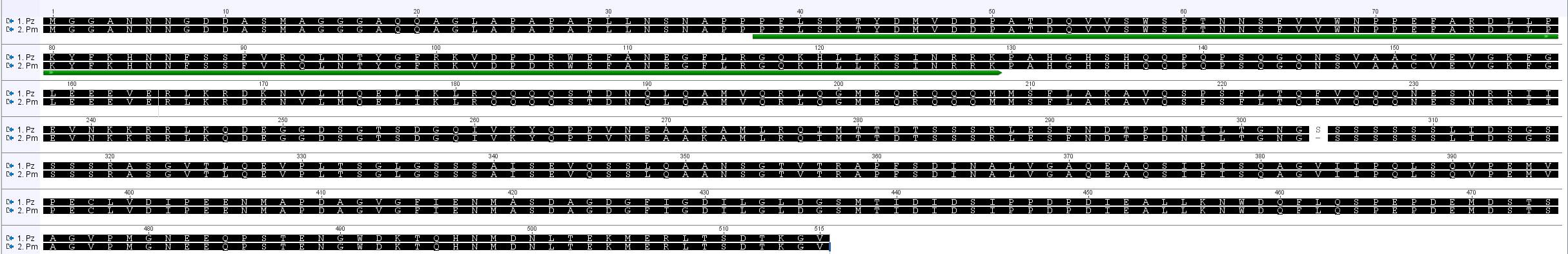


**Fig. S7. Comparative analysis of *PzHSFA1d* and *PmHSFA1d* protein sequences.** The green part is the HSF conserved domain.


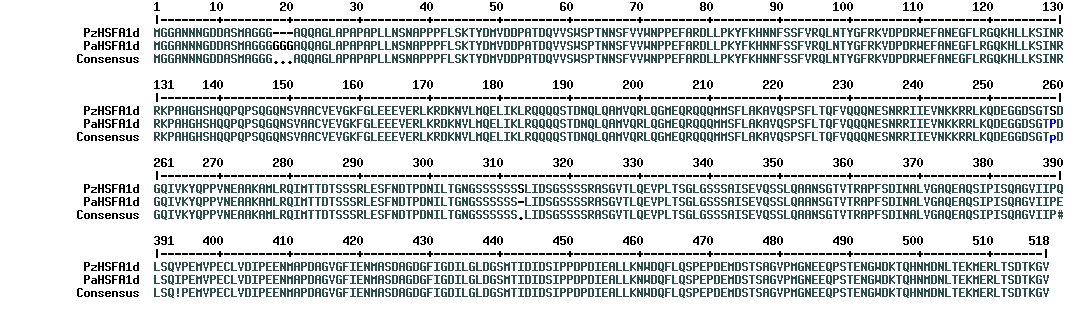


**Fig. S8. Comparison of *HSFA1d* coding sequences of *P. zhengheensis* and apricot.**


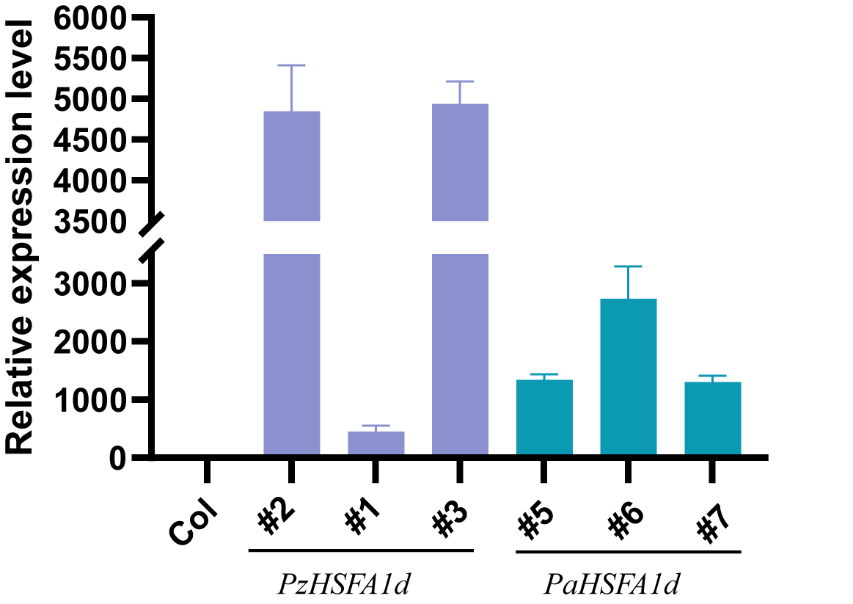


**Fig. S9. The expression levels of *PzHSFA1d* and *PaHSFA1d* were confirmed in transgenic and control *A. thaliana* by RTqPCR amplification**. #1 - #7 repreasented transgenic *A. thaliana* plants.


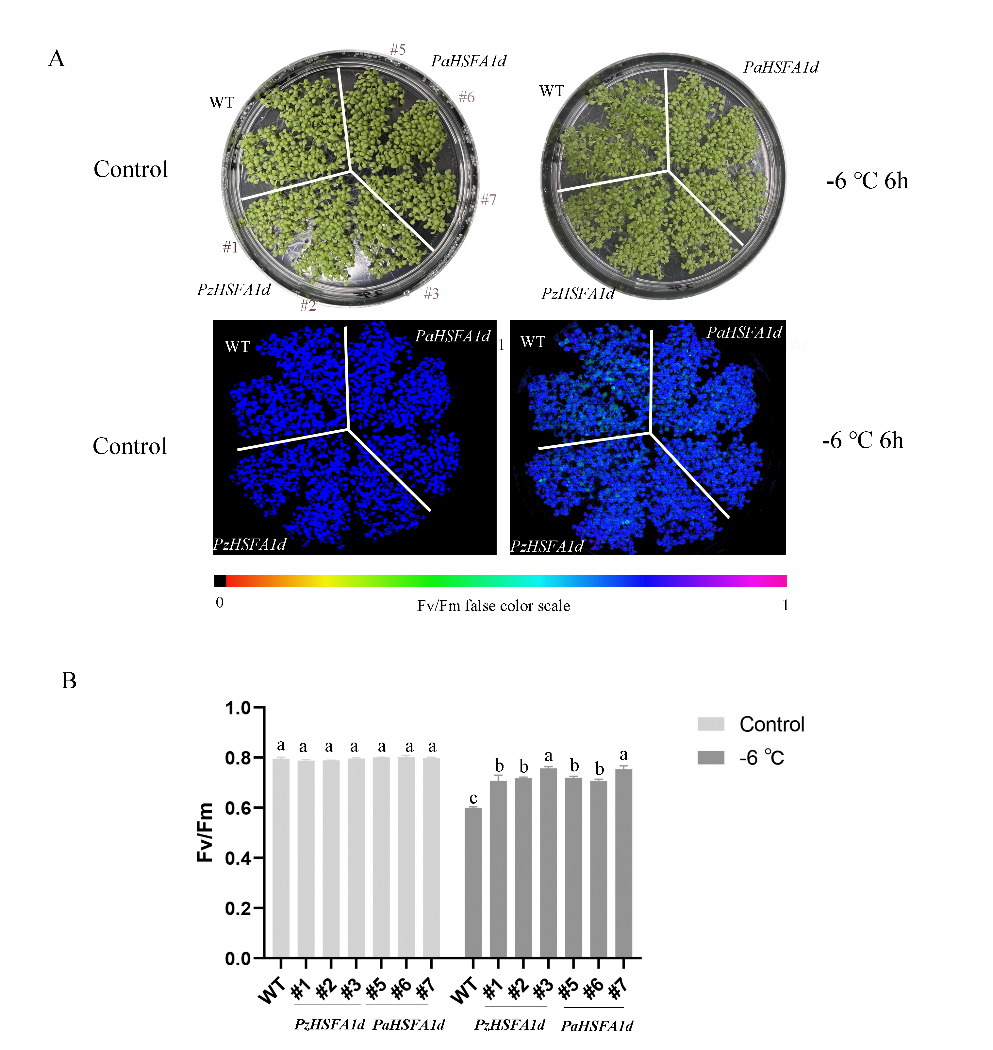


**Fig. S10. Cold treatment of transgenic *A. thaliana*.** (A) Phenotype and chlorophyll fluorescence in cold treatment of transgenic *A. thaliana*. (B) The value of Fv/Fm before and after processing.
