# Supplementary figures and images for: Haplotype-resolved genome of Prunus zhengheensis provides insight into its evolution and low temperature adaptation in apricot
Source: Hortic Res. 2024 Apr 8;11(4):uhae103. doi: 10.1093/hr/uhae103 (PMC11059810; doi:10.1093/hr/uhae103)

A

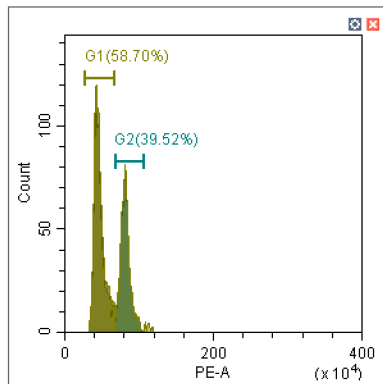

B

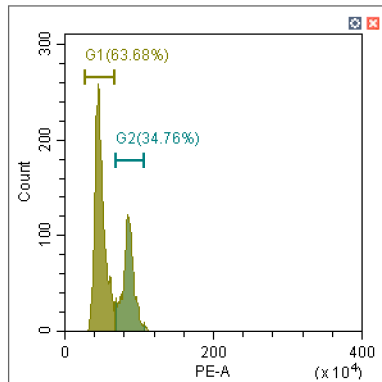

Supplement: Web_Material_uhae103 [file web_material_uhae103.zip › Fig. S1. The genome survey of P. zhengheensis by using flow cytometry.pdf]

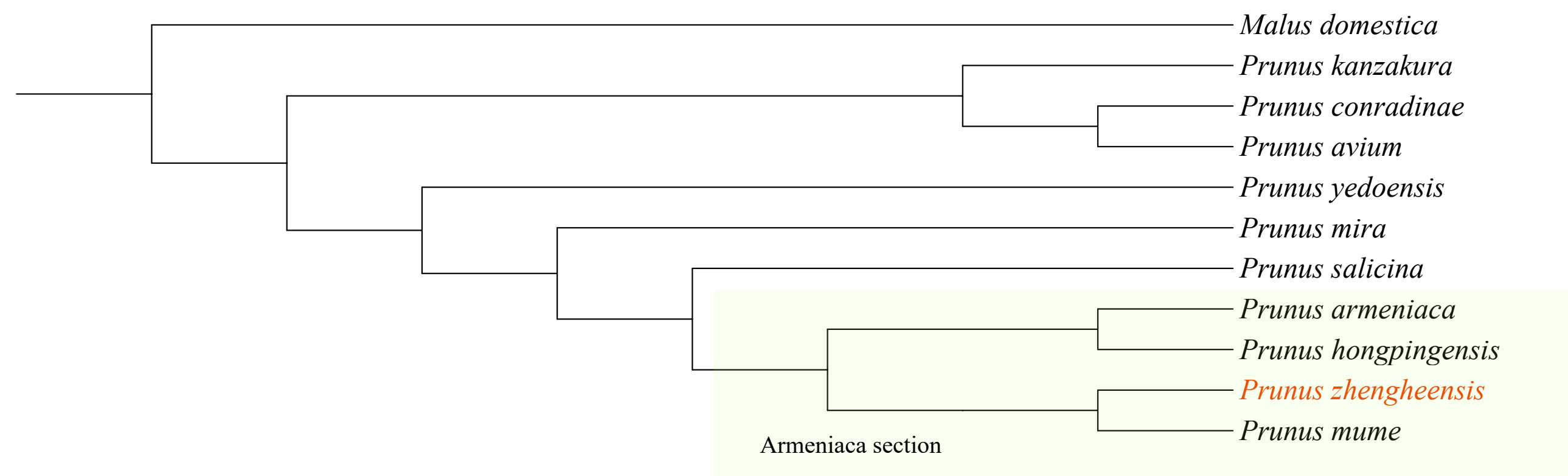

Supplement: Web_Material_uhae103 [file web_material_uhae103.zip › Fig. S2. Phylogenetic reconstruction of P. zhengheensis and other Prunus species based on mitochondrial genomes.pdf]

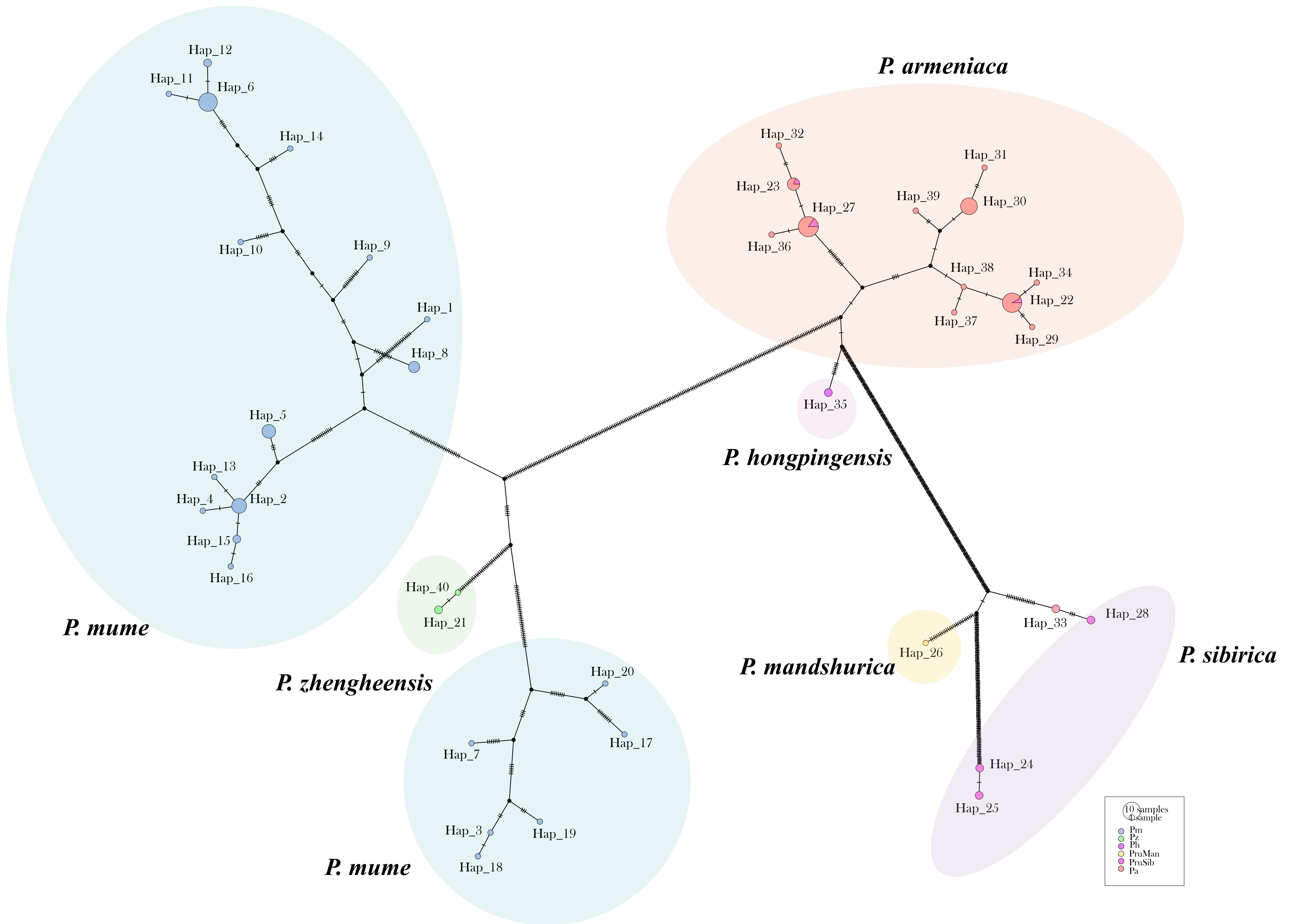

Supplement: Web_Material_uhae103 [file web_material_uhae103.zip › Fig. S3. The genetic relationships among Armeniaca chloroplastic haplotypes are depicted by a median-joining network.pdf]

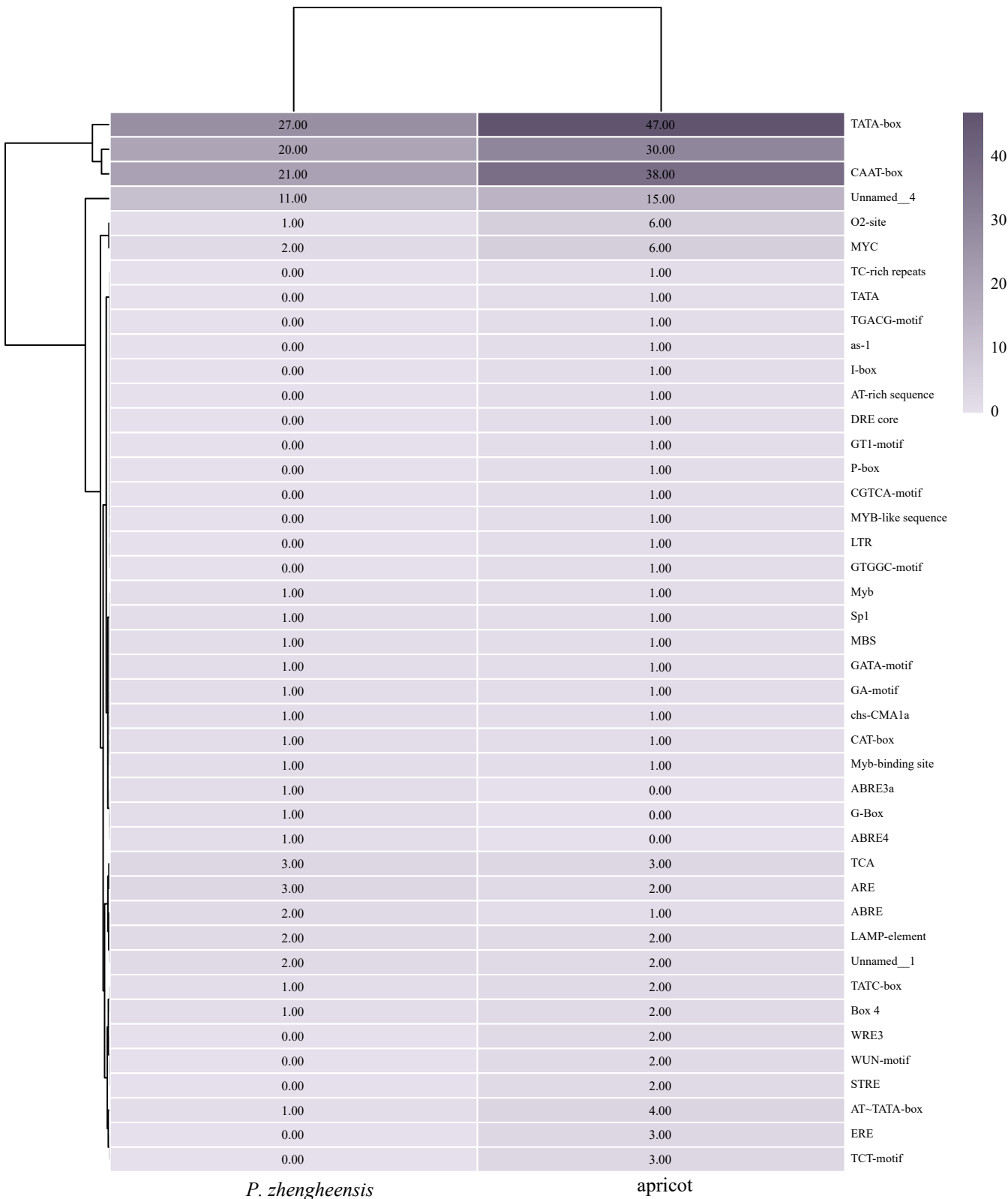

Supplement: Web_Material_uhae103 [file web_material_uhae103.zip › Fig. S5. Promoter cis-acting element analysis..pdf]

# HSFA1d

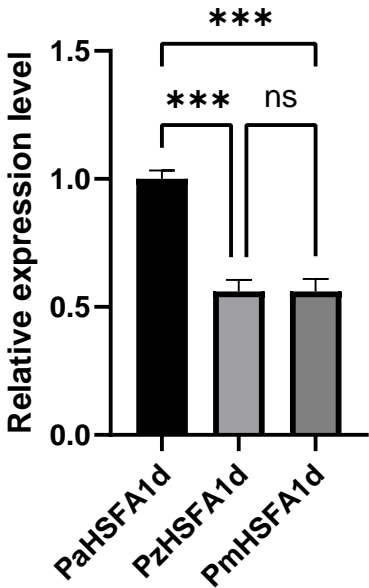

Supplement: Web_Material_uhae103 [file web_material_uhae103.zip › Fig. S6. The expression levels of HSFA1d (PzHSFA1d, PaHSFA1d and PmHSFA1d) gene were confirmed in winter branches by RT-qPCR analysis.pdf]

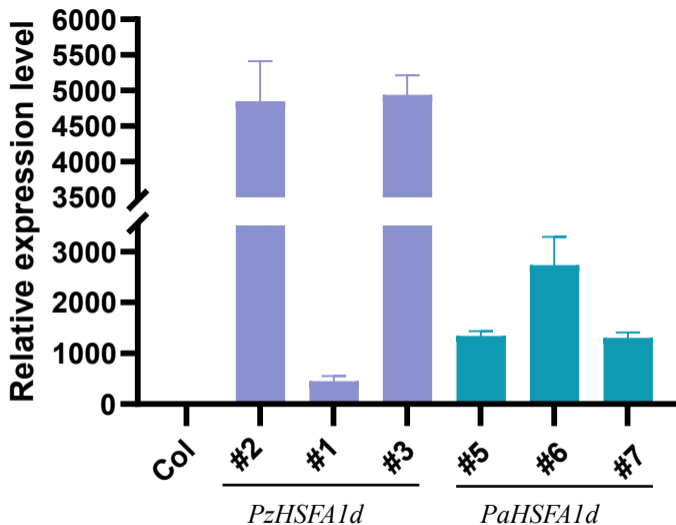

Supplement: Web_Material_uhae103 [file web_material_uhae103.zip › Fig. S9. The expression levels of PzHSFA1d and PaHSFA1d were confirmed in transgenic and control A. thaliana by RTqPCR amplification.pdf]

A

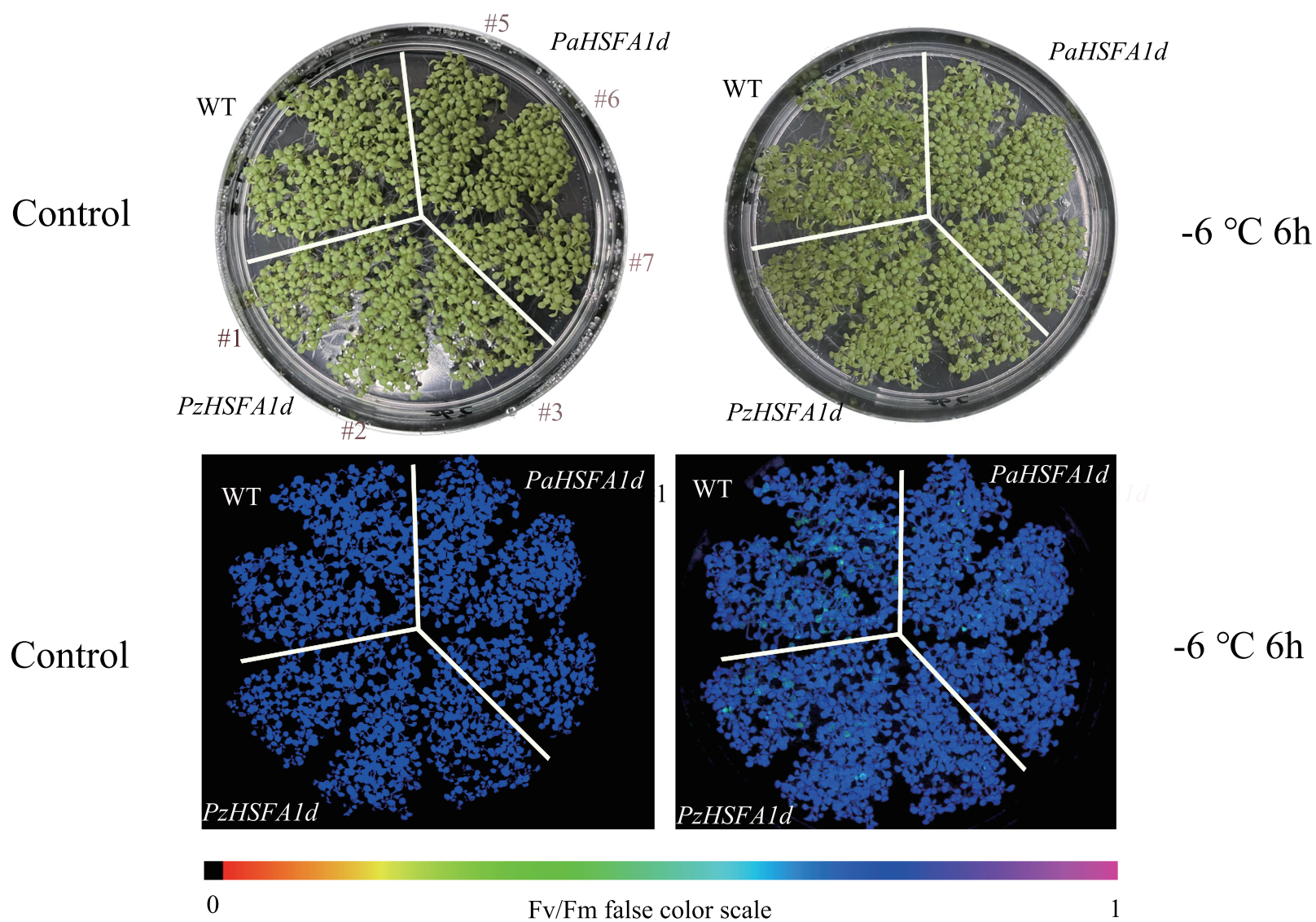

B

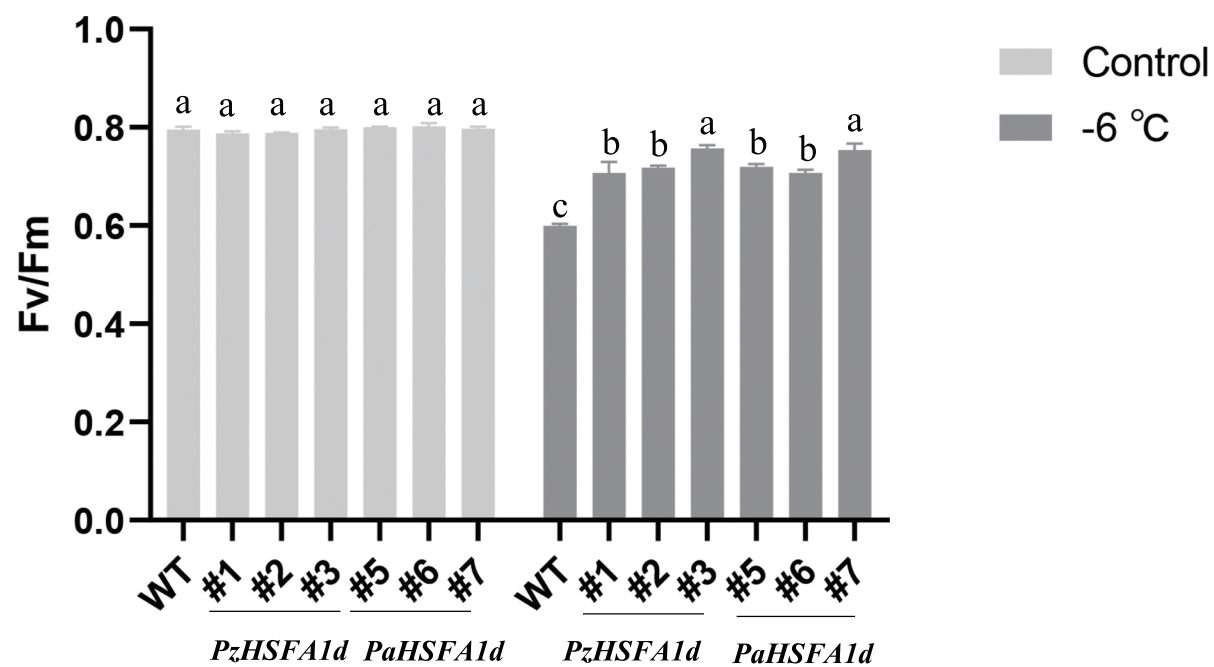

Supplement: Web_Material_uhae103 [file web_material_uhae103.zip › Fig. S10. Cold treatment of transgenic A. thaliana.pdf]
