# Supplementary material for: Haplotype-resolved genome of Prunus zhengheensis provides insight into its evolution and low temperature adaptation in apricot
Source: Hortic Res. 2024 Apr 8;11(4):uhae103. doi: 10.1093/hr/uhae103 (PMC11059810; doi:10.1093/hr/uhae103)
Supplement: Web_Material_uhae103 [file Fig. S4. The sequence comparison between PaHSFA1d and PzHSFA1d promoters.pdf › Fig. S4. The sequence comparison between PaHSFA1d and PzHSFA1d promoters.pdf]

```
            1       10        20        30        40        50        60        70        80        90        100       110       120       130
            |--------+---------+---------+---------+---------+---------+---------+---------+---------+---------+---------+---------+---------|
proPaHSFA1d GTTGAGAGACACCCTAAATAAAATAACATTAAAAAATTAAAACAAAATTAACTCACCCGCTCTACACCCCTCTTCTTGCCAGCCCACCCTTCCACATATCCACCCTGATCTTAACAATCAACACCCTGAA
proPzHSFA1d GTTGAGAAACACCCTAAATAAAATAACATTAAAAAATTAAAACAAAATTAACTCACCCACTCTACACCGCTCTTCTTGCTAGCCCACCCTCCCATAGATCCACCCTGATCTCAACAATCAACACCCTGAA
  Consensus GTTGAGAaACACCCTAAATAAAATAACATTAAAAAATTAAAACAAAATTAACTCACCCaCTCTACACCcCTCTTCTTGCcAGCCCACCCTcCCAcAgATCCACCCTGATCTcAACAATCAACACCCTGAA

            131     140       150       160       170       180       190       200       210       220       230       240       250       260
            |--------+---------+---------+---------+---------+---------+---------+---------+---------+---------+---------+---------+---------|
proPaHSFA1d AAGCTCCCACACAGTCAACAACGTCAAAGTCAAGATCCAACACAAGGAGAGTATTCTGCCAAACCAATAGAGGTCTATCTTTGCTCGCAAGCAGCTCCAGGATGGCTGCACCATCGCCGAGTACAACATC
proPzHSFA1d AAGCTCCCACACAGTCAACAATGTCAAAGTCAAGATCCAACACAAGGAGAGTATTCTGCCAAACCAATAGAGGTCTATCTTTGCTCGCAAGCAGCTCCA---------CACCATCGCCGAGTACAACATC
  Consensus AAGCTCCCACACAGTCAACAAcGTCAAAGTCAAGATCCAACACAAGGAGAGTATTCTGCCAAACCAATAGAGGTCTATCTTTGCTCGCAAGCAGCTCCA.........CACCATCGCCGAGTACAACATC

            261     270       280       290       300       310       320       330       340       350       360       370       380       390
            |--------+---------+---------+---------+---------+---------+---------+---------+---------+---------+---------+---------+---------|
proPaHSFA1d CAAAAATAATCCACTCCCCACCTTGTGCTCTGACTTCGTGGCAGATAGATAGAGTTACCTCTCGTGGGAGTATGTACTAAAAAAAA-TGAAAATGGGAGGAGTTGGAATGGGTTGCTTTATCAAAACATT
proPzHSFA1d CAAAAATAATCCACTCTCCAC-TTGTGCTCTGACTTCGTGGCAGATAGATAGAGTTACCTCTCGTGGGAGGATGTAGTAAAAAAAAATGAAAATGGGAGGAGTTGGAATGGGTTGCTTTATCAAAACATT
  Consensus CAAAAATAATCCACTCcCCAC.TTGTGCTCTGACTTCGTGGCAGATAGATAGAGTTACCTCTCGTGGGAGgATGTAcTAAAAAAAA.TGAAAATGGGAGGAGTTGGAATGGGTTGCTTTATCAAAACATT

            391     400       410       420       430       440       450       460       470       480       490       500       510       520
            |--------+---------+---------+---------+---------+---------+---------+---------+---------+---------+---------+---------+---------|
proPaHSFA1d CCCTGGAGGTCAGAGTCGGTTTATTAGATATGGAGGCTCTGTGCGAAGAAAGCTGATGATGTCCTATATGTATACGTTAAGAATAATGCTACTCTTACTACATTTGTATACCACATTCTCATATCACCTT
proPzHSFA1d CCCTGGAGGT------------------------------------------------------------------------------------------------------------------------
  Consensus CCCTGGAGGT........................................................................................................................

            521     530       540       550       560       570       580       590       600       610       620       630       640       650
            |--------+---------+---------+---------+---------+---------+---------+---------+---------+---------+---------+---------+---------|
proPaHSFA1d ATGTGGCAAATGAGGTGACACATCAATTAAAATTAATTTAAAGTTTTCTTATCTTTTATGATTTATTGACACTTTTTAATTGATGTGATTGTCCACTTCATCTGCCACATAAGGTGGTATGAAAATGTGG
proPzHSFA1d ----------------------------------------------------------------------------------------------------------------------------------
  Consensus ..................................................................................................................................

            651     660       670       680       690       700       710       720       730       740       750       760       770       780
            |--------+---------+---------+---------+---------+---------+---------+---------+---------+---------+---------+---------+---------|
proPaHSFA1d GATATAAATGTGGTAAGTGTAGCATTACTCATAAGTTAAACAACAATTTTTCAAATTTTAAAATTCATAAATCATAAATTAAACCGAAATATGTGGCATCAATTACATTAGGACCCACAAATAGAATCAA
proPzHSFA1d ----------------------------------------------------------------------------------------------------------------------------------
  Consensus ..................................................................................................................................

            781     790       800       810       820       830       840       850       860       870       880       890       900       910
            |--------+---------+---------+---------+---------+---------+---------+---------+---------+---------+---------+---------+---------|
proPaHSFA1d GATCATCCTTCATCTATTTTCACATACTCACATCCATTCAATTTTCTTTTACATACAAAAAAACTGCACATACCCACAAATATAATATAATGTTGGGCTTTTAAAAATATTTGGAGGCTCTTAAATTTTA
proPzHSFA1d ----------------------------------------------------------------------------------------------------------------------------------
  Consensus ..................................................................................................................................

            911     920       930       940       950       960       970       980       990       1000      1010      1020      1030      1040
            |--------+---------+---------+---------+---------+---------+---------+---------+---------+---------+---------+---------+---------|
proPaHSFA1d GGGGCCCTATGCGGTCGCACAGCCCGCACATGCCATGGGCCGACTTTGCTGGAGGTTTGAGAGGTTTTCACCTTGCAACGTTGTGAGAAGAATGAGATGGTCTATGTGGGAAGAAGATGAGGGATAGTTG
proPzHSFA1d --------------------------------------------------------TTGAGAGGTTTTCACCTTGCAACGTTGTGAGAAAAATGAGATGGTCTATGTGGGAAGAAGATGAGGGATAGTTG
  Consensus ........................................................TTGAGAGGTTTTCACCTTGCAACGTTGTGAGAAaAATGAGATGGTCTATGTGGGAAGAAGATGAGGGATAGTTG

            1041    1050      1060      1070      1080      1090      1100      1110      1120      1130      1140      1150      1160      1170
            |--------+---------+---------+---------+---------+---------+---------+---------+---------+---------+---------+---------+---------|
proPaHSFA1d GAACGTGCTTGGGTGGAAGAAGATGAGGGACAGATGATGTAAATTAGAAAAGTTAACAAGGATATTAAAGGCAATTCATTAAAATTCATTAAACAACCCGGTTCCTTGTTTAGCTTTTCGTGAAAGCTGA
proPzHSFA1d GAACGTGCTTGGGTGGAAGAAGATGAGGAATAGATGATGTAAATTAGAAAAGTTAACAAGGATATTAAAGGCAATTCATTAAAATTCATTAAACAACCCGATTCCTTGTTTAGCTTTCCGTGAAAGCTGA
  Consensus GAACGTGCTTGGGTGGAAGAAGATGAGGaAcAGATGATGTAAATTAGAAAAGTTAACAAGGATATTAAAGGCAATTCATTAAAATTCATTAAACAACCCGaTTCCTTGTTTAGCTTTcCGTGAAAGCTGA

            1171    1180      1190      1200      1210      1220      1230      1240      1250      1260      1270      1280      1290      1300
            |--------+---------+---------+---------+---------+---------+---------+---------+---------+---------+---------+---------+---------|
proPaHSFA1d AATAAAATTAGCCCTTAAGCTGCTTTCTAAAATCACGATCGCTGACAGCTATTTTATCCCAAAAGTGATTATCTCGCATTTTACCAAACAATGTGAACTTCAATAACTTTATCAAAATTCACTTTTTAGC
proPzHSFA1d AATAAAATCAGCCCTTAAGCTGCTTTCTAAAATCACGGTCGCTGGCAGCTATTTTATCCCAAATGTGATTATCTTGCATTTTACCAAGCAAGGTGAACTTCAATAACTTTATCAAAAATCACTTTTTAGC
  Consensus AATAAAATcAGCCCTTAAGCTGCTTTCTAAAATCACGaTCGCTGaCAGCTATTTTATCCCAAAaGTGATTATCTcGCATTTTACCAAaCAAgGTGAACTTCAATAACTTTATCAAAAaTCACTTTTTAGC

            1301    1310      1320      1330      1340      1350      1360      1370      1380      1390      1400      1410      1420      1430
            |--------+---------+---------+---------+---------+---------+---------+---------+---------+---------+---------+---------+---------|
proPaHSFA1d CCAAATAATCAATGCTAAACGGGCACTAAATTGAACAACAGTAGATGATAAAACTTCTTGCCACTGGAAGATTGATAAATTTTGATAGAGATGATGTCATTTGTAATGGGTTTGTATCAATATATTGATG
proPzHSFA1d CCAAATAATCAATGCTAAACGGGCACTAAATCGAACAACAGTAGATGATAAAACTTCTTGCCACTGGAAGATTGATAAATTTTGATAGAGATGATGTCATTTGTAATGGGTTTGTATCAGTATATTGATG
  Consensus CCAAATAATCAATGCTAAACGGGCACTAAATcGAACAACAGTAGATGATAAAACTTCTTGCCACTGGAAGATTGATAAATTTTGATAGAGATGATGTCATTTGTAATGGGTTTGTATCAaTATATTGATG

            1431    1440      1450      1460      1470      1480      1490      1500      1510      1520      1530      1540      1550      1560
            |--------+---------+---------+---------+---------+---------+---------+---------+---------+---------+---------+---------+---------|
proPaHSFA1d AAATATAATAGTAAATGGTGATAATAGATAAATAATTTCAAGTGTTCAAAATTTATATTAAAATTTAT-TATGTGAT-------GATTTATGCGGTTTTAAAAAGACTACATTGAATTGAATCTATGTAT
proPzHSFA1d AAATATAATAGTAAATGGTGATAATAGATAAATAATTTCAAGTGTTCAAAATTTATATTAAAATTTGTATATGTGATTTGGTTTGATTTATGCGGTTCTAAAAAGACTACACTGAACTGAATCTATGTAT
  Consensus AAATATAATAGTAAATGGTGATAATAGATAAATAATTTCAAGTGTTCAAAATTTATATTAAAATTTaT.TATGTGAT.......GATTTATGCGGTTcTAAAAAGACTACAcTGAAcTGAATCTATGTAT

            1561    1570      1580      1590      1600      1610      1620      1630      1640      1650      1660      1670      1680      1690
            |--------+---------+---------+---------+---------+---------+---------+---------+---------+---------+---------+---------+---------|
proPaHSFA1d ATAAAGCAAAAGGCAGAAAATGGTGAAACATTCAAAATATCAGAAAATATCATTGGTTAATTCAAACATTAAGAATTGAAATTATTAATTAAATTAATTAAATGAAGATAATATGGTAAATTCATATTTT
proPzHSFA1d ATAAAGCAAAAGACAGAAAATGGTGAAACATTCAAAATATCAAAAAATGATATTAGTTAATTCAAACATTAAGAATTGAAATTATTAATTAAAT---------GAAGATAATATGGTAAATTCACATTTT
  Consensus ATAAAGCAAAAGaCAGAAAATGGTGAAACATTCAAAATATCAaAAAATaacATTaGTTAATTCAAACATTAAGAATTGAAATTATTAATTAAAT.........GAAGATAATATGGTAAATTCAcATTTT

            1691    1700      1710      1720      1730      1740      1750      1760      1770      1780      1790      1800      1810      1820
            |--------+---------+---------+---------+---------+---------+---------+---------+---------+---------+---------+---------+---------|
proPaHSFA1d TTTCATATTAAAAAAAATTAAAATTTAAAATTAAAAAAAAAAAAAGAGATATTAGATCTTACTTTTATAGAACATAACTATCAATATTATCTTTCCTTAATTTTAAAAATGAAAAAATGAAAAAAATAAA
proPzHSFA1d TTTCATATTTAAAAAAAATTAAAATTAAAA--------ACAAAATGAGATATTATATCCTACTTTTATAGAACATAACTATCAATATTATCTTTCCTTAATTCTAAAAAT---------TAAAAAATTAA
  Consensus TTTCATATTaAAAAAAAaTaAAAaTTAAAA........AaAAAAaGAGATATTAgATCcTACTTTTATAGAACATAACTATCAATATTATCTTTCCTTAATTcTAAAAAT.........aAAAAAATaAA

            1821    1830      1840      1850      1860      1870      1880      1890      1900      1910      1920      1930      1940      1950
            |--------+---------+---------+---------+---------+---------+---------+---------+---------+---------+---------+---------+---------|
proPaHSFA1d ACCAATTAGCACATAGTTGAGGGAGGCTAGTCAAAATAATTATTGTTCTTCTGATCCGGTTCAAGATATAATTTTTTTT-AAGTAAAAAAGTCATTTAATTAACATGTTGAAATTTTTTCTAATTTTCAT
proPzHSFA1d ACCAATTAGCACATACGTGAAG-AGGCTAGTCAAACTAATTATTGTTCTTCTGATCCGGTTCAAGATATAATTTTTTTTTAAGTAAAAAAGTCATTTAATTAACATGTTGAAATTTTTTCTAATTTTCAT
  Consensus ACCAATTAGCACATAcgTGAaG.AGGCTAGTCAAAaTAATTATTGTTCTTCTGATCCGGTTCAAGATATAATTTTTTTT.AAGTAAAAAAGTCATTTAATTAACATGTTGAAATTTTTTCTAATTTTCAT

            1951    1960      1970      1980      1990      2000      2010      2020      2030      2040      2050      2060      2070      2080
            |--------+---------+---------+---------+---------+---------+---------+---------+---------+---------+---------+---------+---------|
proPaHSFA1d TATTTTCCAAAATTCGAGGACATCATCATCCATCTCCCAGAGAGGAGGCAACTGTTCGTTCCCGTTTCGTGCGTCGGTGTCCGCCCTTTTATACACCTTCCCTACTCTTCGCCTAATCCCACTGTCCGTA
proPzHSFA1d TATTTTCCTAAATTCGATGACATCATCATCCATCTCCCAGAGAGGAGGCAACTGTTCGTTCCCGTTTCGTGCGTCGGTGTCCGCCCTTTTATACACCTTCCCTACTCTTCGCCTAATCCCACTGTCCGTA
  Consensus TATTTTCCaAAATTCGAgGACATCATCATCCATCTCCCAGAGAGGAGGCAACTGTTCGTTCCCGTTTCGTGCGTCGGTGTCCGCCCTTTTATACACCTTCCCTACTCTTCGCCTAATCCCACTGTCCGTA

            2081    2090      2100      2110      2120      2130      2140      2150      2160      2170      2180      2190      2200      2210
            |--------+---------+---------+---------+---------+---------+---------+---------+---------+---------+---------+---------+---------|
proPaHSFA1d CACAACACACACACTCTCTCTCTCTCT----------GATAGAGGCAGTCTAGGGTTTTTGAGCTAAGAGCTCTCTTCCGTACCATAGAAATGGCCTCTGCAAAACCCTAGCCAAGCCAATAATCCCCAT
proPzHSFA1d CACAACACACACACTCTCTCTCTCTCTCTCTCTCTCTGATAGAGGCAGTCTAGGATTTTTGAGCTAAGAGCTCTCTTCCGTACCATAGAAATGGCCTCTGCAAAACCCTAGCCAAGCCAATAATCCCCAT
  Consensus CACAACACACACACTCTCTCTCTCTCT..........GATAGAGGCAGTCTAGGaTTTTTGAGCTAAGAGCTCTCTTCCGTACCATAGAAATGGCCTCTGCAAAACCCTAGCCAAGCCAATAATCCCCAT

            2211    2220      2229
            |--------+--------|
proPaHSFA1d TTCTGTGCCATTATTTTAA
proPzHSFA1d TTCTGTGCCATTATTTTAA
  Consensus TTCTGTGCCATTATTTTAA
```
